# Magnetic transferrin nanoparticles (MTNs) assay as a novel isolation approach for exosomal biomarkers in neurological diseases

**DOI:** 10.1186/s40824-023-00353-2

**Published:** 2023-02-16

**Authors:** Yoon Ok Jang, Hee-Sung Ahn, Thuy Nguyen Thi Dao, JeongYeon Hong, Wangyong Shin, Young-Min Lim, Sun Ju Chung, Jae-Hong Lee, Huifang Liu, Bonhan Koo, Myoung Gyu Kim, Kyunggon Kim, Eun-Jae Lee, Yong Shin

**Affiliations:** 1 Department of Biotechnology, College of Life Science and Biotechnology, Yonsei University, Seoul, 03722 Republic of Korea; 2 Department of Convergence Medicine, Asan Medical Center, Seoul, 05505 Republic of Korea; 3 Asan Institute for Life Sciences, Asan Medical Center, Seoul, 05505 Republic of Korea; 4 Department of Biomedical Sciences, University of Ulsan College of Medicine, Seoul, 05505 Republic of Korea; 5 Department of Neurology, Asan Medical Center, University of Ulsan College of Medicine, Seoul, 05505 Republic of Korea

**Keywords:** Brain-derived blood exosome, Neurological disease, Extracellular vesicle, Magnetic nanoparticle, Liquid chromatography − mass spectrometry

## Abstract

**Background:**

Brain-derived exosomes released into the blood are considered a liquid biopsy to investigate the pathophysiological state, reflecting the aberrant heterogeneous pathways of pathological progression of the brain in neurological diseases. Brain-derived blood exosomes provide promising prospects for the diagnosis of neurological diseases, with exciting possibilities for the early and sensitive diagnosis of such diseases. However, the capability of traditional exosome isolation assays to specifically isolate blood exosomes and to characterize the brain-derived blood exosomal proteins by high-throughput proteomics for clinical specimens from patients with neurological diseases cannot be assured. We report a magnetic transferrin nanoparticles (MTNs) assay, which combined transferrin and magnetic nanoparticles to isolate brain-derived blood exosomes from clinical samples.

**Methods:**

The principle of the MTNs assay is a ligand-receptor interaction through transferrin on MTNs and transferrin receptor on exosomes, and electrostatic interaction via positively charged MTNs and negatively charged exosomes to isolate brain-derived blood exosomes. In addition, the MTNs assay is simple and rapid (< 35 min) and does not require any large instrument. We confirmed that the MTNs assay accurately and efficiently isolated exosomes from serum samples of humans with neurodegenerative diseases, such as dementia, Parkinson's disease (PD), and multiple sclerosis (MS). Moreover, we isolated exosomes from serum samples of 30 patients with three distinct neurodegenerative diseases and performed unbiased proteomic analysis to explore the pilot value of brain-derived blood protein profiles as biomarkers.

**Results:**

Using comparative statistical analysis, we found 21 candidate protein biomarkers that were significantly different among three groups of neurodegenerative diseases.

**Conclusion:**

The MTNs assay is a convenient approach for the specific and affordable isolation of extracellular vesicles from body fluids for minimally-invasive diagnosis of neurological diseases.

**Supplementary Information:**

The online version contains supplementary material available at 10.1186/s40824-023-00353-2.

## Background

As lifespan prolongs, neurodegenerative diseases, such as dementia, Parkinson’s disease (PD), and multiple sclerosis (MS), become more prevalent and serious threats to human well-being [1]. The progressive worsening of neurodegenerative diseases over time is because neurons, once damaged, rarely recover since their majority are post-mitotic cells [2]. Moreover, there are currently no treatments to reverse the brain damage. Disease biomarkers are essential for early diagnosis and sensitive detection of neurological deterioration.

Proteins in the blood are a good candidate for such biomarkers because neurons and glial cells are composed of numerous proteins that are released into the blood upon brain damage [3, 4]. They are especially desired for diagnostics because of their high accessibility and convenience. However, it is difficult to reliably gauge these proteins with conventional detection methods because of their very low concentrations in the blood. The single-molecule array has been suggested as a recent cutting-edge technology that can detect trace amounts of blood protein [5]; however, it can only be applied to a limited number of proteins. Moreover, although blood proteins sensitively reflect neuronal damage, these biomarkers, such as neurofilament light chain (NfL) and glial fibrillary acidic proteins, are not specific because their levels can increase due to various neurological conditions [4, 6–8]. Another consideration is the possible direct interaction of proteins with other blood proteins, such as degrading enzymes [9], which raises the concern that these proteins may not accurately reflect the status of the brain.

Extracellular vesicles (EVs) are lipid-bound vesicles released by cells into the extracellular space [10, 11]. EVs play crucial roles in the regulation of biological processes, including intercellular communications, transportation of complex cargo, disease progression, and immunity regulation [12–14]. The subtypes of EVs are exosomes (30 − 200 nm), microvesicles (100 − 1000 nm), and apoptotic bodies (1000 − 5000 nm), which are differentiated based on their biogenesis, size, function, content, and release pathway [15, 16]. Exosomes enter the circulatory system and are found in biological fluids, including blood [17], saliva [18], synovial fluid [19], urine [20], semen, sputum, breast milk [21], and cerebrospinal fluid (CSF). These EVs and their biologically active cargos may offer prognostic information on a broad range of physiological processes, such as central nervous system communication [22], tissue repair [23], immune responses [24], stem cell maintenance [25], and pathological processes in neurodegenerative diseases [26], chronic inflammation [27], cardiovascular diseases [28], cancer [29], and lipid metabolic diseases [30]. Furthermore, exosomes facilitate the exchange of substances and information between cells and have been used as promising sources of disease biomarkers and tumor vaccines on account of their unique contents, such as proteins, lipids, miRNAs, and mRNAs [31]. It has also been demonstrated that the surfaceome of exosomes can be used for the noninvasive diagnosis of pancreatic cancer [32]. Therefore, exosomes in body fluids, including blood, may be potentially used as minimally invasive early diagnostic biomarkers.

Proteins contained in blood EVs may merit as biomarkers because EV envelopes can protect proteins from interacting with other proteins and being degraded, thereby enabling increased protein content concentrations, stability, and reliability [9]. Accordingly, some promising results have suggested that EV proteins successfully reflect the status of several neurodegenerative diseases [33–35]. However, in most studies, only some target proteins were selected and analyzed. Given the tremendous number of proteins in the brain, picking up several proteins in EVs may have caused unintended bias. Proteomic analysis approaches may maximize the value of EVs as biomarkers; mass spectrometry-based proteomics, which allows measuring as many blood proteins as possible to identify new molecular biomarkers and facilitate the discovery of disease signatures for brain disorders [36–39], may potentially offer more prospects of EV as a biomarker for neurodegenerative diseases. In addition, attempts to evaluate blood EV proteins as biomarkers have been made in only a single disease entity without evaluating them in terms of specific biomarkers that discriminate among various neurodegenerative diseases. Although EV proteins may reflect neurodegenerative diseases, it is not yet known whether they show distinct patterns for each disease or exhibit common features upon brain damage regardless of causes. Therefore, it would be remarkable to evaluate EV proteomes from various neurodegenerative diseases simultaneously.

The commonly used EV isolation methods include ultracentrifugation (UC) [40], polymer-based precipitation [41], size-based filtration [42], microfluidic-based isolation [43], immune affinity-based technique, and size-exclusion chromatography [44]. However, these methods are limited by various factors that require expensive and scaled-up equipment [45] and a purification step to remove impurities before analysis. Additionally, they are time-consuming, laborious procedures prone to EV trapping and EV membrane clogging [46], resulting in relatively low purity and recovery of EVs [47]. Therefore, precise isolation and purification of EVs (exosomes) are critically important for discovering or validating biomarkers for disease diagnostics. However, despite the promising potential of EVs as carriers of biomarkers for diagnostic purposes in clinical trials [48–51], there is currently no standardized method for the optimal isolation and purification of brain-derived blood exosomal biomarkers in neurological diseases.

Hence, we report a magnetic transferrin nanoparticles (MTNs) assay to isolate brain-derived blood exosomes in neurological diseases. To accurately isolate and purify exosomes, transferrin and magnetic nanoparticles (MNPs) were combined to create the MTNs. Transferrin was chosen as a ligand because it can bind to the transferrin receptor on the surface of exosomes [51, 52]. MNPs have also attracted much attention as promising sources for exosome isolation due to the advantages of precise manipulation of particles and small-volume capacity. The principle of the MTNs assay is a ligand − receptor interaction through transferrin − transferrin receptor interaction and electrostatic interaction between positively charged MNPs and negatively charged exosomes to overcome the limitations of the traditional isolation methods, which are laborious, time-consuming, and dependent on expensive devices. Exosome isolation using the MTNs assay is simple and rapid, does not require any antibody or centrifugation, and can be automatically or manually performed in a short time (< 35 min). We confirmed that the MTNs assay accurately and efficiently isolated exosomes from colorectal cancer cell culture medium (CCM) and human serum samples by quantitatively and qualitatively comparing the isolated exosomes with those isolated via the existing assays. In addition, we hypothesized that proteomic profiles of exosome proteins of neurodegenerative diseases with different mechanisms would differ from each other. Using the MTNs assay, we isolated the brain-derived blood exosomes from the serum samples of 30 patients with neurodegenerative diseases, such as dementia, PD, and MS, and performed unbiased proteomic analysis to explore the pilot value of 746 exosomal protein profiles as biomarkers by using liquid chromatography − mass spectrometry (LC–MS). From the results of the principal component analysis (PCA), principal component 1 (PC1) distinguished the PD group from the MS and dementia groups, suggesting that the levels of these differentially abundant proteins (DAPs) in serum exosomes can be used to distinguish neurodegenerative diseases. Principal component 2 (PC2) clearly differentiated the MS and dementia groups. Finally, we found a significant difference in 21 brain-derived blood exosomal biomarkers among the three groups from a comparative statistical analysis. Consequently, we demonstrated the applicability of the MTNs assay to isolate the brain-derived blood exosomes, thereby providing a convenient approach for rapid and affordable isolation of clinically applicable exosomes from body fluids for the minimally-invasive diagnosis of neurological diseases.

## Materials and Methods

### Synthesis and characterization of MTNs

For the synthesis of Fe_3_O_4_@SiO_2_ MNPs, 29 mL of Igepal® CO-520 (238,643, Sigma-Aldrich, St. Louis, MO, USA) was ultrasonically dispersed in 71 mL cyclohexane (#179,191, Sigma-Aldrich) for 10 min at room temperature (RT). Then, 100 mg of Fe_3_O_4_ (#637,106, Sigma-Aldrich) MNPs were added to the prepared solution. Ammonia solution (NH_4_OH; 28%) and tetraethyl orthosilicate (TEOS, 98%, #333,859, Sigma-Aldrich) were added dropwise to the reaction mixture and stirred for 24 h at RT for hydrolysis and condensation of the silica precursor. Next, (3-aminopropyl)triethoxysilane (APTES, 99%, #440,140, Sigma-Aldrich) was added dropwise to the mixture containing Fe_3_O_4_@SiO_2_ MNPs, and the mixture was stirred for 24 h at RT. The Fe_3_O_4_@SiO_2_-NH_2_ MNPs were collected by magnetic separation, washed several times with ethanol, and re-dispersed in 100 mL ethanol. To synthesize Fe_3_O_4_@SiO_2_@transferrin MNPs, 2 mL glutaraldehyde (GA; #G6257, Sigma-Aldrich) was added to the solution containing Fe_3_O_4_@SiO_2_-NH_2_ MNPs, which was stirred for 1 h at RT. Then, 10 mg of transferrin (#T0665, Sigma-Aldrich) was added to the solution and stirred for 24 h at RT. After the supernatant was discarded, the modified MTNs were collected by magnetic separation and were washed several times with ethanol. Finally, the synthesized MTNs were dispersed in 10 mL ethanol and separated into 10 tubes (10 mg of particles in 1 mL for each reaction). The synthesized MTNs were immediately used or stored at 4 °C.

The surface morphology and size distribution of the Fe_3_O_4_-MNPs and synthesized MTNs were characterized using scanning electron microscopy (SEM; Hitachi S-4700, Japan). Approximately 1 mL solutions of Fe_3_O_4_-MNPs and MTNs were prepared. Approximately 20 µL of Fe_3_O_4_-MNPs and MTNs suspensions were dropped onto a silicon substrate, and the surfaces were dried in a fume hood at RT overnight. After Pt coating, the dried suspensions were placed in the SEM analysis chamber for imaging. The zeta potentials of Fe_3_O_4_-MNPs and synthesized MTNs were examined using a NanoBrook ZetaPlus System (90Plus PALS, Brookhaven Instruments Corp., USA). Approximately 1 mL solutions of Fe_3_O_4_-MNPs and MTNs were prepared in ethanol. The solutions were diluted at the ratio of 1:400 with distilled water (DW) before zeta potential analysis at 25 °C. All measurements were performed in triplicate. Fourier-transform infrared (FTIR) spectroscopy (Vertex 70 FTIR spectrometer, Bruker, Germany) was performed on pure Fe_3_O_4_-MNPs and modified MTNs to obtain information on the chemical modifications. Functional groups of pure Fe_3_O_4_-MNPs and modified MTNs were identified by ultraviolet − visible (UV–Vis) spectrophotometry (Cary 100 UV–Vis spectrophotometer, Varian, Inc.)

### EV isolation

Colorectal carcinoma (HCT-116; ATCC CCL-247) cells were cultured in Dulbecco's modified Eagle medium (DMEM, Life Technologies, Carlsbad, CA, USA) supplemented with 10% exosome-depleted fetal bovine serum (FBS, #A2720803, Thermo Fisher Scientific, Pittsburgh, PA, USA) and 1% antibiotic–antimycotic (Gibco, USA) and maintained in a humidified incubator at 37 °C under 5% CO_2_. For the isolation of EVs, HCT-116 cells were grown to approximately 80% confluence, and HCT-116 CCM cells were collected by centrifugation at 400 g for 30 min at 4 °C. The supernatant was collected and filtered using a hydrophilic polyvinylidene fluoride (PVDF) membrane (0.22 µm; GVS, Italy). The supernatant was immediately used for experiments or stored at − 20 °C for up to 4 weeks.

### Participants

We have prospectively recruited patients with neurological disorders who visited the Department of Neurology at the Asan Medical Center since June 2018, and collected their blood samples in case of informed consents. Blood from these patients was drawn into a serum-separating tube, followed by centrifugation at 2,500 g for 10 min at 4 °C according to standard procedures [53]. The supernatants were transferred to a new microtube and stored at − 80 °C until used. Clinical information such as Expanded Disability Status Scale (EDSS), Hoehn − Yahr (HY) stage, and Global Deterioration Scale (GDS) was also investigated at the time of blood sampling. Among these patients, patients with MS (n = 10), idiopathic PD (n = 10), and dementia (n = 10) but those without other diseases were selected and analyzed. Sample size was determined to be 10 for each disease; we selected this small sample number to rapidly explore the feasibility of our novel EV method and its performance for disease diagnosis. The diseases were diagnosed according to the criteria specified for each disease: MS by the 2017 McDonald criteria [54], idiopathic PD by the MDS criteria [55], and dementia by the DSM 5 criteria [56], respectively. All the patients with MS had relapse-remitting MS in the remission phase with a median EDSS of 4.0. Patients with PD had a median HY stage of 2.5, and those with dementia demonstrated a median GDS of 4. None of the patients with MS or PD demonstrated cognitive impairment, which affects activities of daily living. Detailed patient information is shown in Supplementary Table S1. This study was reviewed and approved by the Ethics Committee of the Institutional Review Board of the AMC and previously described procedure [57]. All the experimental procedures were performed in accordance with the guidelines of the Institutional Review Board of the AMC (IRB No. 2020–0297, 2018–0653).

### EV isolation assays

UC and total exosome isolation (TEI) methods were used to conduct a comparative study with the MTNs. The HCT-116 CCM was pre-cleared by centrifugation at 400 g for 30 min at 4 °C, and the resulting supernatant was passed through a 0.22 μm PVDF filter via syringe. For EV isolation using UC, 20 mL of prepared CCM was ultracentrifuged at 110,000 g (SW28 rotors, Beckman Coulter, Brea, CA, USA; and PA rotors, Hitachi ultracentrifuge) for 70 min at 4 °C. The supernatant was discarded, and then the pellets were resuspended in 200 µL or 1 mL of phosphate-buffered saline (PBS) for SEM, transmission electron microscopy (TEM), nanoparticle tracking analysis (NTA), and zeta potential assays. Otherwise, the pellets were lysed with 200 µL of RIPA buffer for western blotting (WB) and LC–MS/MS.

For EV isolation using the TEI reagent (#4,478,359, Invitrogen), 20 mL of prepared CCM was added to 10 mL of TEI reagent, which was vortexed to obtain a homogenous solution. The mixture was incubated at 4 °C overnight, followed by centrifugation at 10,000 g at 4 °C for 1 h. Subsequent steps were described in the previous paragraph.

For EV isolation using the MTNs, 20 mL of prepared CCM was thoroughly mixed with MTNs (10 mg per reaction) using a rotator at RT for 30 min. The MNPs and CCM were separated using a magnet stand, and then the MNPs were washed twice with PBS. The PBS was discarded, and the MNPs were added to 200 μL or 1 mL of elution buffer (10 mM NaHCO_3_, pH 10.6) for SEM, TEM, NTA, and zeta potential assays. Otherwise, the pellets were lysed as described above in this section. The isolated EVs were immediately used or stored at − 80 °C.

For EV isolation from clinical specimens using the MTNs, 500 µL of human serum samples were thoroughly mixed with MTNs (10 mg per reaction) using a rotator at RT for 30 min. The serum and MNPs were separated using a magnet stand, and the MNPs were washed twice with PBS. Then, the PBS was discarded, and the MNPs were added to 200 µL of RIPA buffer. After incubation, the lysed EVs were collected in a new tube. The isolated EVs were immediately used for experiments or stored at − 80 °C.

### Characterization of EVs

The morphology of the isolated EVs was characterized using SEM and TEM. The concentration of EVs was analyzed using NTA, the surface charges of EV were determined using a NanoBrook ZetaPlus, and the protein constituents were identified through WB. The purity of the isolated EVs was determined as the ratio of the nanovesicle count (via NTA) to the protein quantification via a Bradford assay.

For the SEM analysis, EVs were isolated using MTNs, UC, and the TEI reagent and were then fixed with 4% paraformaldehyde. Subsequently, the EVs were diluted in DW, pipetted onto silicon chips, and dried under a ventilation hood, after which a coating of Pt was applied by sputtering. Images were captured using the SEM (Hitachi S-4700).

For the TEM analysis, the immunogold staining antibodies of specific proteins were used to identify exosomal proteins and verify the existence of exosomal surface proteins likely to interact with transferrin. In this study, CD63 (exosome marker) and CD71 (transferrin receptor) markers were confirmed by TEM. Briefly, isolated EVs were dropped onto the formvar/carbon-coated copper grids and incubated at 37 °C for 30 min. Then, the grids were blocked with 5% bovine serum albumin (BSA) at RT for 20 min, then washed with PBS. For immunogold labeling of CD63 or CD71, the grids were incubated with anti-CD63 antibody or anti-CD71 monoclonal antibody, respectively, at 4 °C overnight. Next, the grids were washed with PBS and incubated with anti-mouse IgG conjugated to 10 nm gold particles or with anti-rabbit IgG conjugated to 10 nm gold particles at RT for 1 h. The grids were washed and fixed in 2.5% GA for 5 min before being contrasted with 3% lead citrate solution for 5 min. Images were acquired by TEM (JEM-F200, JEOL, Japan).

For NTA experiments, EVs isolated using the different isolation assays (MTNs, UC, and TEI reagent) were diluted at the ratio of 1:100, 1:200, or 1:400 in PBS. The size distribution and concentration of isolated EVs were determined via NTA (NanoSight NS300, Malvern Instruments, UK). Surface charges of EVs were assessed using a NanoBrook ZetaPlus. All isolated EV samples were diluted in DW (pH 7.0).

For WB, isolated EVs were analyzed. The WB analysis of specific proteins was used to confirm the purity of the isolated EVs: CD71 (transferrin receptor on exosome surface); CD63, CD9, and CD81 (belongs to the tetraspanin family and is an exosome-specific protein); GRP78 (a member of the heat shock protein family of molecular chaperons associated with the endoplasmic reticulum (ER) and cellular stress and found in apoptotic bodies); ADP-ribosylation factor 6 (ARF6), which is a microvesicle protein marker); GM130 (Golgi marker); calnexin (ER marker). Chemiluminescence signals were detected with a ChemiDoc XRS (Bio-Rad). The WB was performed using a previously described procedure [58].

### LC–MS analysis

For peptide sample preparation, exosome protein samples (1 μg/μL concentration) were dissolved in 200 μL of SDS lysis buffer (5% SDS, 50 mM TEAB pH 8.5). After adding dithiothreitol to a final concentration of 20 mM to the denatured sample, it was incubated at 95 °C for 10 min. The chemically reduced sample was then placed in iodoacetamide at a final concentration of 40 mM and reacted for 30 min at 25 °C in the dark. With a final concentration of 1.2% phosphoric acid, acidified samples were attached to suspension-trapping (S-Trap) mini columns (#CO2-mini-80, ProtiFi, Farmingdale, NY, USA). Following the manufacturer's protocol, we performed S-Trap proteolysis by adding 8 μg of Lys-C/trypsin mixture (#V5071, Promega, Madison, WI, USA) and incubating at 37 °C for 16 h (HaileMariam et al., 2018). The digested peptide mixture was freeze-dried with a cold trap (CentriVap Cold Traps; Labconco, Kansas City, MO, USA) and stored at − 80 °C until use.

HPLC-grade acetonitrile and water were purchased from Avantor (Radnor, PA, USA). LC–MS-grade formic acid (FA) was purchased from Thermo Fisher Scientific. Dried digested peptide samples were reconstituted in 0.1% FA, and the total peptide concentrations were measured using a UV–Vis spectrophotometer (NanoDrop One, Thermo Fisher Scientific) at a wavelength of 280 nm, with the sample type option set to "1 Abs = 1 mg/mL." The resuspended sample was adjusted to a concentration of 1 μg/μL, and 5 μL was injected into a C18 PepMap™ trap column (20 mm × 100 µm i.d., 5 µm, 100 Å; Thermo Fisher Scientific) and separated by an Acclaim™ PepMap 100 C18 column (500 mm × 75 µm i.d., 3 µm, 100 Å; Thermo Fisher Scientific) over 200 min (250 nL/min) at 50 °C. Mobile phase A was 0.1% FA and 5% DMSO in water, and mobile phase B was 0.1% FA, 5% DMSO, and 80% acetonitrile in water. A gradient of 5–40% B for 150 min, 40–95% for 2 min, 95% for 23 min, 95–5% for 10 min, and 5% for 15 min was applied. The liquid chromatography system was coupled to a Q Exactive mass spectrometer (Thermo Fisher Scientific) with a nano-electrospray ionization (ESI) source. Selected ion spectra were acquired in a data-dependent mode with an automatic switch between a full scan and top 20 fragment ion scans. The automatic gain control target value for the selected ion spectra was 3 × 10^6^ with a maximum injection time of 100 ms at a resolution of 70,000 at *m/z* 400. The automatic gain control ion target for the fragment ion was set to 1 × 10^6^ with a maximum injection time of 50 ms at a resolution of 17,500 at *m/z* 400. Repeated peptides were dynamically excluded for 20 s. All mass spectrometry data were measured once per sample and were deposited in the PRIDE archive (www.ebi.ac.uk/pride/archive/projects/PXD038555) [59].

### Identification and quantification of proteome data

Raw files of tandem mass spectrometry (MS/MS) spectra were retrieved against the UniProtKB/Swiss-Prot human protein sequence database (March 2021) [60] by the SEQUEST HT embedded in Proteome Discoverer (version 2.4; Thermo Fisher Scientific). Search parameters were set at 10 ppm tolerance for precursor ion mass and 0.02 Da for the fragmentation mass. The trypsin peptides toleration was set at up to two false cleavages, carbamidomethylation of cysteines was set as fixed modification, and N-terminal acetylation and methionine oxidation were set as variable modifications. The false discovery rate (FDR) was calculated using the target-decoy search strategy, and the peptides within 1% of the FDR were selected using the post-processing semi-supervised learning tool Percolator [61] based on the SEQUEST result. Label-free quantitation of proteins was calculated using the precursor ion peak intensity for peptides in proteins with two or more unique or razor peptides, excluding those with methionine oxidation.

### Statistical analysis

Raw data were analyzed by Perseus software (version 1.6.15.0) [62]. For the comparative statistical analysis, protein selection criteria were based on having a quantified value in at least seven samples from three groups: dementia (*N* = 10), PD (*N* = 10), and MS (*N* = 10). Log_2_-transformed raw data were normalized by the width adjustment method. Normalized proteomic data were subjected to mean-centering correction per protein for the 30 samples to correct the batch effect [63, 64]. The mean-wise batch correction was used in two batches. Batch 1 consisted of five patients with PD, five patients with dementia, and five patients with MS; and batch 2 consisted of five patients with PD, five patients with dementia, and five patients with MS; based on sample preparation date [65]. Sample groups were compared by ANOVA with Benjamini − Hochberg FDR correction. Perseus software was used for 1) hierarchical clustering with both Euclidean distance and complete linkage and 2) the PCA. Results were visualized using RStudio (version 1.3.1093), a component of R software (version 3.6.0). Other software packages included ggplot2 for drawing boxplots and scatter plots. Data were analyzed by one-way ANOVA with Tukey's HDS post hoc test using SPSS software version 24.0 (IBM Corp., Armonk, NY, USA). For all analyses, *P* < 0.05 were considered statistically significant.

### Gene ontology (GO) analysis

The pathway enrichment analysis was performed by the FunRich 3.1.3 software [66] and EnrichR [67]. DAPs in the three groups were analyzed using the ClueGO (version 2.5.4) [68] plugin for Cytoscape (version 3.6.1) [69]. To group GO terms, the kappa score was set at 0.4, and the number of overlapping genes to combine groups was 50%. To find the neuronal-related proteins, we searched DAPs in the Synaptic Gene Ontologies (SYNGO) [70], which is an empirical, experienced scientist-curated resource with synapse function and gene enrichment studies and other gene sets of elevated brain genes in the Human Protein Atlas (HPA) [71].

## Results

### Principle of the MTNs

**Fig. 1:**
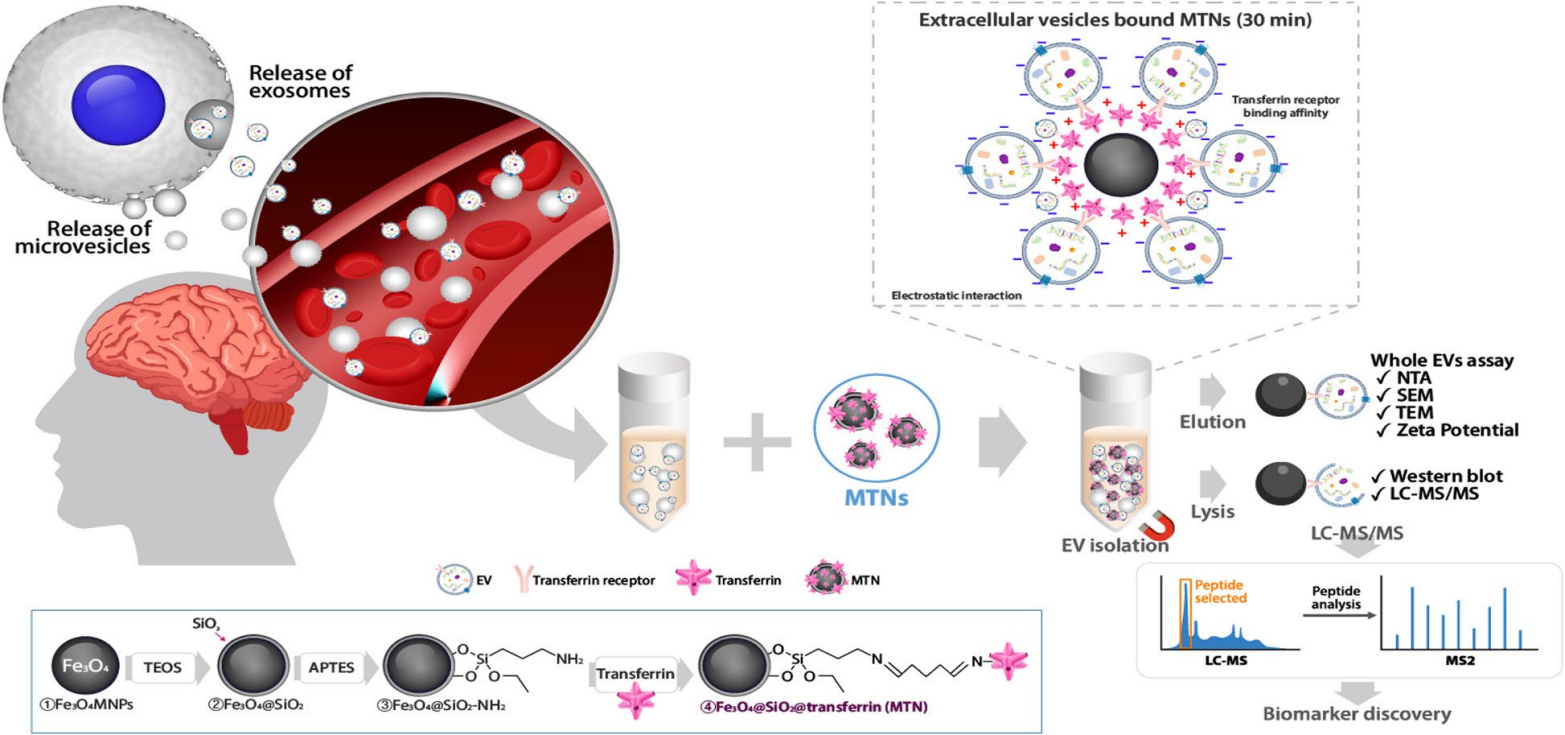
Schematic illustration and application of the MTNs assay to EV isolation. Schematic diagram of the EV isolation from human serum and colon cancer CCM using MTNs. Schematic diagram of the synthesis of MTNs (below rectangle). LC–MS/MS schematic part was prepared by BioRender (http://app.biorender.com).

The synthesis of MTNs and the principle of their application for the isolation and detection of EVs are illustrated in Fig. 1. MTNs were synthesized by conjugation of transferrin with the Fe_3_O_4_-NPs. The synthesized MTNs can bind to exosomes through the ligand − receptor interaction of the transferrin on the MNPs and the transferrin receptor on the exosomes. In addition, the MTNs can also bind to exosomes via electrostatic interaction of the positively charged surface of the MTNs and the negatively charged surface of the exosomes. Both interactions enable strong and selective capture of the exosomes. Accordingly, we assessed whether the MTNs could be used to isolate brain-derived blood exosomes from serum samples of humans with neurological diseases. Brain-derived blood exosomes were first captured by MTNs, which were then magnetically isolated and washed to realize the isolation and purification of exosomes in 35 min without any large instrumentation. Captured exosomes were eluted for whole EV assays, such as NTA, SEM, TEM, and zeta potential analysis. Additionally, captured EVs were lysed to study their molecular contents, such as proteins, via WB and LC–MS/MS analysis to identify candidate exosomal protein biomarkers.

### Characterization of the MTNs

**Fig. 2:**
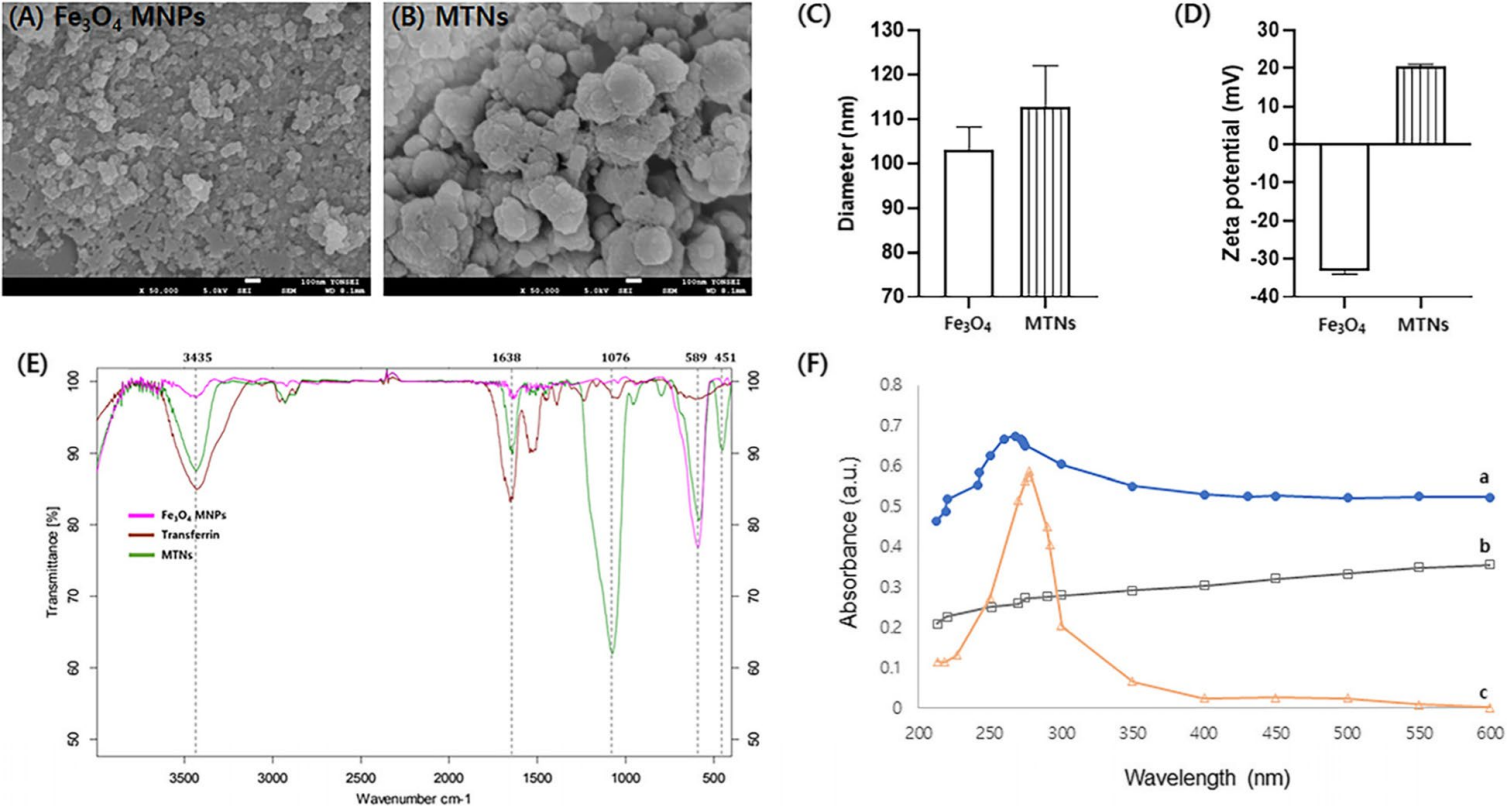
Characterization of the synthesized MTNs. SEM images of (**A**) Fe_3_O_4_-MNPs and (**B**) MTNs. **C** Size distributions of Fe_3_O_4_-MNPs and MTNs. **D** Zeta-potentials of Fe_3_O_4_-MNPs and MTNs. **E** and **F** Analysis of the molecular structures of the synthesized MTNs via FTIR and UV–Vis. (**E**) FTIR spectra of the materials; Fe_3_O_4_-MNPs (pink line), transferrin (brown line), and MTNs (green line). **F** UV–Vis spectra of the synthesized MTNs (curve a), Fe_3_O_4_-MNPs (curve b), and pure transferrin (curve c).

For the synthesis of MTNs, we performed several characterization experiments (Supplementary Fig. S1). To synthesize the Fe3O4@SiO2-NH2-transferrin nanoparticles (MTNs), we used the previously described protocol to study systematically for the amine-functionalization of the Fe3O4@SiO2-NH2 magnetic nanoparticles (MNPs) [28]. Then, we modified some protocol to enhance the efficiency of the synthesis. We examined whether the washing buffer had any influence on the Fe3O4@SiO2-NH2 MNPs either with ethanol or distilled water. We observed that the Fe3O4@SiO2-NH2 MNPs washed with ethanol were clearer than those washed with distilled water (DW). Thus, the Fe3O4@SiO2-NH2 MNPs were washed several times with ethanol (Supplementary Fig. S1a). Then, we tested various transferrin concentrations and determined the optimal concentration via the zeta potential analysis (Supplementary Fig. S1b). Next, we examined the yield of synthesized Fe3O4@SiO2-NH2 and MTNs at two incubation times with transferrin (3 or 24 h) using HCT-116 cell culture medium and human normal serum sample. After isolated the EVs (exosomes), we tested with Coomassie blue staining and western blot analysis (CD63 and actin) (Supplementary Fig. S1c-d). The results of both incubation times were not significantly different. Thus, 24 h was chosen as the optimal incubation time. Based on the characterization results, the surface morphology and uniform size distribution of the pure Fe_3_O_4_-MNPs and synthesized MTNs were identified in SEM images (Fig. 2a − c). The average sizes of the pure Fe_3_O_4_-MNPs and synthesized MTNs were around 103.14 ± 5.02 and 112.84 ± 9.1 nm, respectively (Fig. 2c), which indicated the uniform distribution of the MNPs. Figure 2d shows the zeta potentials of the Fe_3_O_4_-MNPs and synthesized MTNs. The surface coatings of the pure Fe_3_O_4_-MNPs and synthesized MTNs had charge values of –33.27 ± 0.8 and 20.4 ± 0.58 mV, respectively. The zeta potential results indicate that synthesized MTNs can be used as evidence for the isolation of negatively charged EVs by electrostatic interaction. FTIR spectroscopy was used to identify the chemical bonding and molecular structure of the materials. The FTIR spectra of pure Fe_3_O_4_-MNPs, transferrin, and synthesized MTNs are shown in Fig. 2e, respectively. The strong absorption peak at 589 cm^−1^ assigned to Fe–O stretching vibrations from iron oxide was observed for pure Fe_3_O_4_-MNPs and synthesized MTNs [72]. The FTIR spectrum of transferrin showed absorption peaks at 1638 cm^−1^ (amide I), 1527 cm^−1^ (amide II), and 3435 cm^−1^ (stretching vibrations of O − H). The synthesized MTNs showed absorption peaks at 589 cm^−1^ (Fe–O stretching vibrations), 1638 cm^−1^ (amide I), and 3435 cm^−1^ (stretching vibrations of O–H). Moreover, synthesized MTNs showed additional absorption peaks at 1076 cm^−1^ (asymmetric stretching bonds of Si–O–Si) and 451 cm^−1^ (Si–O–Si or O–Si–O bending modes) [73]. These FTIR results indicate that transferrin was successfully attached to the MTNs through cross-linking. The characterization of the pure Fe_3_O_4_-MNPs, transferrin, and synthesized MTNs by UV–Vis spectroscopy is shown in Fig. 2f. The UV–Vis spectrum of pure Fe_3_O_4_-MNPs showed no characteristic absorbance peaks (Fig. 2f, curve a). Meanwhile, transferrin showed a peak in the UV region at the wavelength of 278 nm (Fig. 2f, curve b). The MTNs also showed a peak at the wavelength of 268 nm (Fig. 2f, curve c), which indicated that transferrin was successfully coated on Fe_3_O_4_-MNPs.

### Validation of the isolated exosomes using MTNs assay

**Fig. 3:**
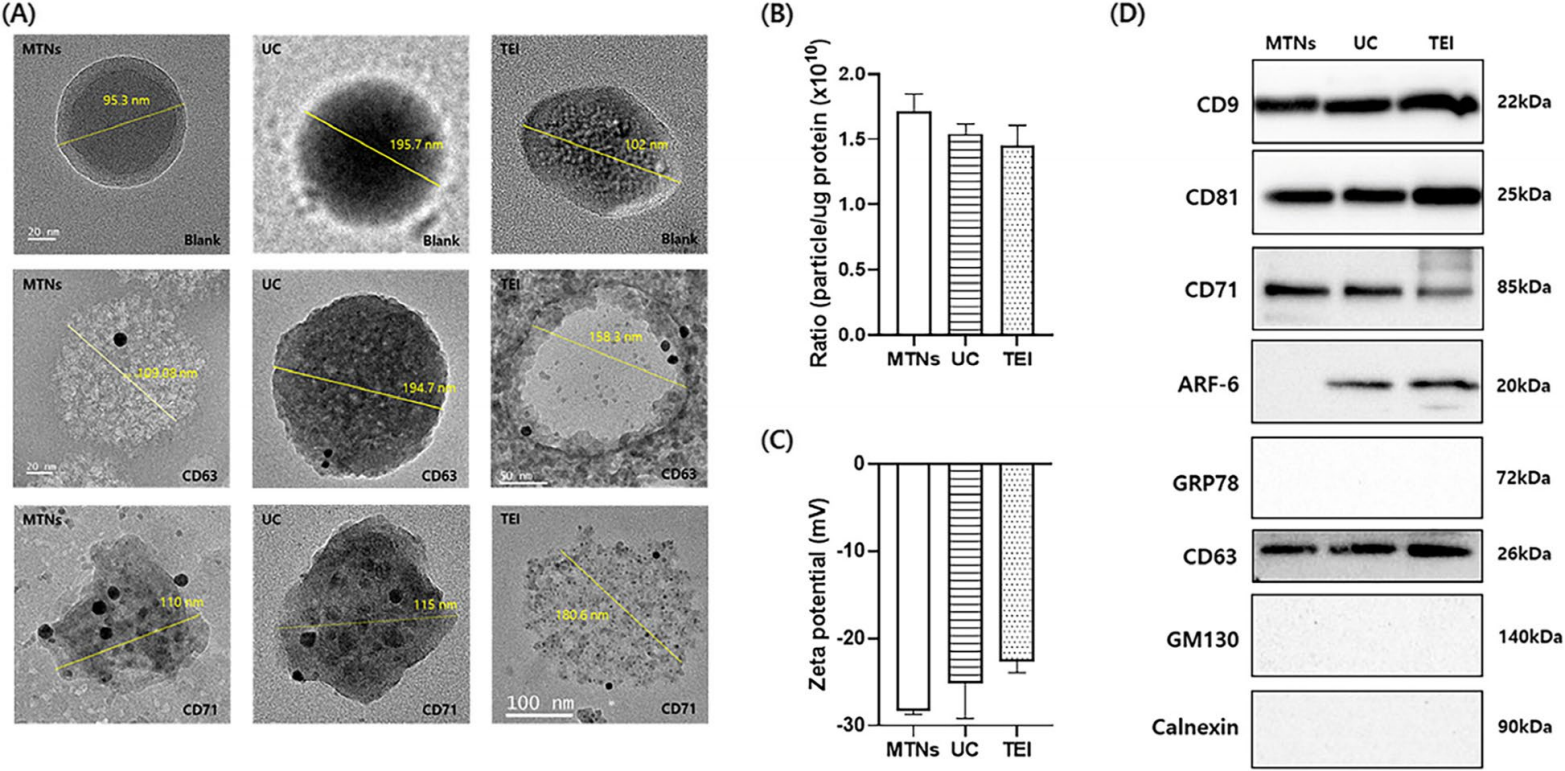
Validation of exosome isolation. **A** Representative TEM image of the exosomes isolated from colon cancer CCM using MTNs, UC, or TEI. TEM micrographs of exosomes were revealed by immunogold CD63-labeling and CD71-labeling. Blank (no labeling) corresponds to the exosomes isolated using the three different isolation methods. **B** Purity of EVs (exosomes), based on the ratio of EV (exosome) particle concentration using the NTA to protein concentration measured using the Bradford assay. **C** Zeta potential of the exosomes isolated from colon cancer CCM using MTNs, UC, or TEI. **D** WB analysis of exosome-specific markers (CD9, CD81, and CD63), transferrin receptor marker (CD71), microvesicle marker (ARF6), and apoptotic-body marker (GRP78), GM130, and calnexin. Lanes 1 − 3: The exosomes were isolated using MTNs (lane 1), UC (lane 2), and TEI (lane 3).

EVs from HCT-116 CCM were isolated using MTNs, UC, and TEI assays (Fig. 3 and Supplementary Fig. S2). Overall, EV isolation by MTNs took less time (~ 35 min for the binding reaction) than the TEI reagent (overnight reaction) and UC (~ 75 min for multiple steps of centrifugation). The EVs isolated from HCT-116 CCM using MTNs, UC, and TEI assays were visualized by SEM and TEM. The SEM images revealed round-shaped EVs with a size range of 30–200 nm (Supplementary Fig. S3). These results were substantiated by the TEM findings (Fig. 3a).

All three groups of isolated EVs were further characterized by immunolabeling against CD63 and CD71 and imaged by TEM. We observed round-shaped particles with the morphology of exosomes after immunolabeling against CD63 and CD71 (Fig. 3a). Furthermore, CD71 was analyzed to verify the binding of transferrin with transferrin receptors on the exosomal membrane. The exosomes were characterized by size range, morphology, and exosome-specific protein (CD63). The size range, morphology, exosome-specific protein (CD63), and transferrin receptor (CD71) verified that exosomes were successfully isolated using the MTNs assays.

The EVs isolated from HCT-116 CCM using MTNs, UC, and the TEI reagent were also identified using NTA (Supplementary Fig. S2). The average concentration (particles/mL) of EVs isolated by MTNs, UC, and TEI assays was 1.71 ± 0.137, 1.543 ± 0.73, and 1.458 ± 0.149 (× 10^10^), respectively (Fig. 3b). The average concentration of EVs isolated by the MTNs assay was fold higher than when isolated by UC and TEI assays. However, exosome aggregation might affect the concentration and average size of EVs during NTA measurement.

The zeta potential of the EVs isolated by MTNs, UC, and TEI assays was assessed to evaluate their integrity and stability, which revealed negative zeta potentials of –36.20 ± 1.19, –25.13 ± 4.09, and –22.67 ± 1.27 mV, respectively (Fig. 3c). The zeta potentials of the isolated EVs using MTNs assay were in the range of –28 to –36 mV (Supplementary Fig. S3) due to their plasma membrane structure, thereby suggesting their good stability in solution. Exosomes have specific proteins (including CD63, CD9, and CD81) on their surface that are recognized by specific antibodies. We examined the EV purity by immunoblotting for marker proteins specific to EVs isolated from HCT-116 CCM. CD9, CD63, and CD81 are exosome-specific markers, CD71 is a transferrin receptor marker, GRP78 is an ER-localized protein (indicates apoptotic bodies), and ARF6 is a microvesicle-related protein. The expressions of CD63, CD9, CD81, and CD71 were found in the proteins of EVs isolated by MTNs, UC, and TEI assays (Fig. 3d). Although ARF6 was found in the EV proteins isolated using UC and TEI assays (low intensity), it was not found in the EV proteins isolated using the MTNs assay. This indicated that very few amounts of microvesicles were bound to MTNs. Moreover, for all three isolation techniques, negative results were observed for GRP78, which indicated that the EV samples did not contain apoptotic bodies, and GM130 or calnexin, which indicated that the EV samples did not contain Golgi or ER. These data provide conclusive evidence that representative EVs, in terms of their biochemical and physical properties, can be isolated using the MTNs assay. The results indicate that MTNs can be used to isolate exosomes with high purity from CCM.

### Brain-derived blood exosome proteins using MTNs assay

**Fig. 4:**
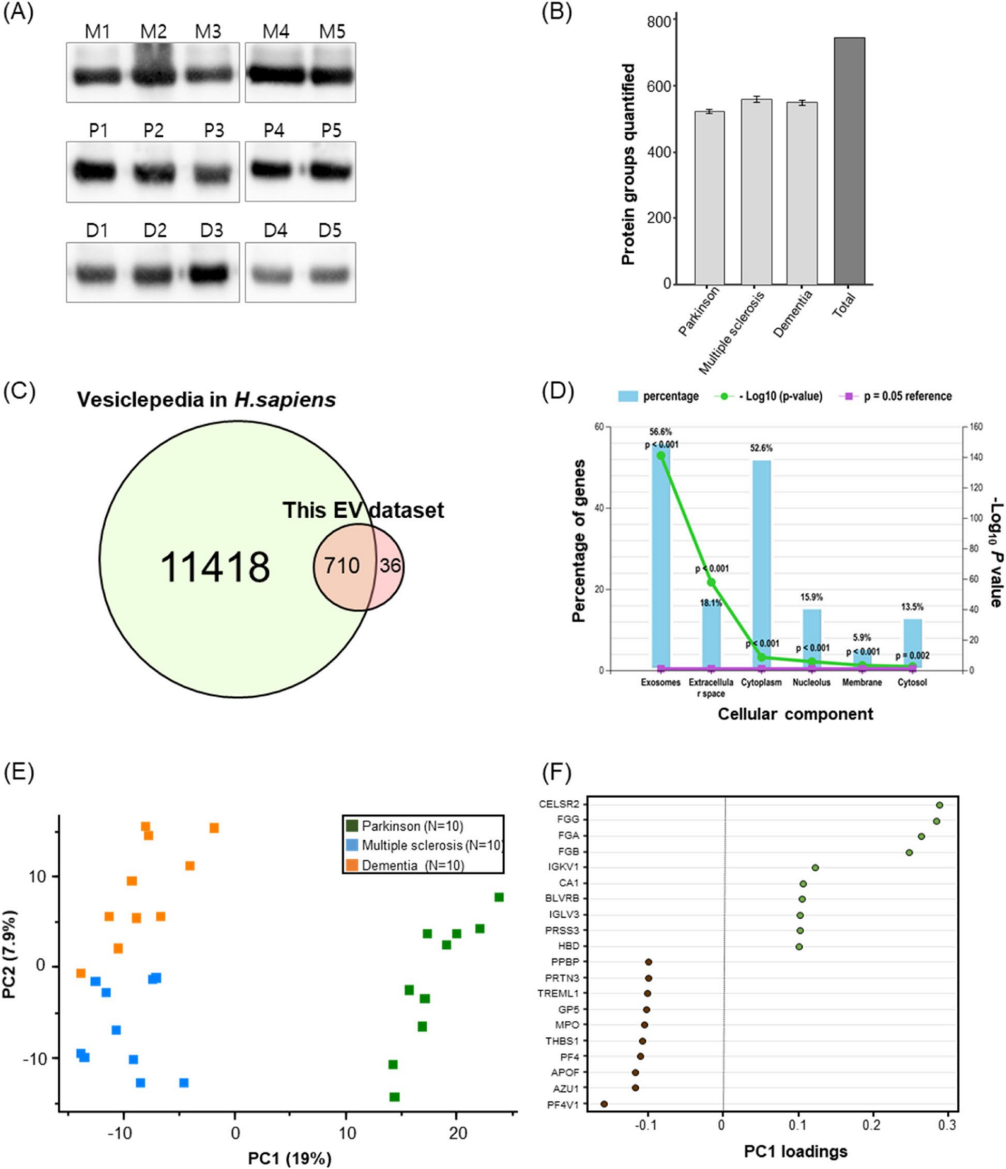
Characterization and multiple dimension reduction of plasma exosome proteins. **A** Representative western blots of the CD63 protein in 15 serum exosome samples among the three groups **B** The number of quantified proteins in the three sample groups (**C**) Venn diagram of proteins between Vesiclepedia in *Homo sapiens* and this study result. **D** Cellular component of GO of quantified proteins in FunRich (version 3.1.3). **E** Principal component analysis of the three groups by the proteins. **F** The loadings of the top 10 and bottom 10 proteins of principal component 1. NB: The three groups are P: Parkinson’s disease (*N* = 10); M: multiple sclerosis (*N* = 10); D: dementia (*N* = 10).

To determine the applicability of MTNs for the isolation of exosomes from clinical specimens, we applied the assay to 30 human serum specimens from patients with neurological diseases. The exosomes obtained from the human serum samples were isolated within 35 min, and the isolated exosomes were then identified by WB using CD63, an exosome-specific marker. The expression of CD63 was found in the exosomal proteins isolated by MTNs from the specimens (Fig. 4a). Then, the serum-derived exosomes were analyzed for the assessment of constituent proteins via single LC–MS/MS runs. A total of 746 proteins were quantified in at least one sample in 30 LC–MS/MS analyses (Supplementary Table S2). The mean (standard deviation) number of quantified proteins across the three groups was similar; PD (524 ± 6.4), MS (560 ± 25.7), and dementia (549 ± 24.9) (Fig. 4b). Most of these proteins were annotated in Vesiclepedia (95.17%) [74] (Fig. 4c). In FunRich [66], these proteins were mainly located in the exosome (56.6%, *P* < 0.001) and extracellular space (18.1%, *P* < 0.001; Fig. 4d). Statistical analysis was performed on 550 proteins by selecting proteins, considering missing values, estimating missing values, performing normalization, and correcting for batch effects (Supplementary Table S3 and Supplementary Fig. S4a-c). The distribution according to the order of average protein abundance was shown, with the proteins being mapped by curated exosomal proteins and top 100 most cited proteins from Vesiclepedia and brain-elevated proteins from HPA (Supplementary Fig. S5a, b) and additionally annotated with subcellular location and tissue specificity in UniProt (Supplementary Table S4). PCA of PD, dementia and MS apparently grouped them in PC1 (19% explained variance) and PC2 (7.9% explained variance) (Fig. 4e). Based on the loading values of the proteins contributing to PC1, the top 10 (CELSR2, FGG, FGA, FGB, IGKV1, CA1, BLVRB, IGLV3, PRSS3, and HBD), high in PD, and bottom 10 (PPBP, PRTN3, TREML1, GP5, MPO, THBS1, PF4, APOF, AZU1, and PF4V1) proteins, low in PD, were displayed (Fig. 4f) and had significant quantitative differences from the rest (*P* < 0.05), except for one, PRSS3, which was significantly different from MS (*P* < 0.05) but not dementia (Supplementary Fig. 6). In detail, an extensive neurodegenerative disease-related literature search for the above PC1-highly contributing 20 proteins was executed with public resources (Supplementary Table S5). CELSR2, which had the highest PC1 loading in PD, is known to be a negative regulator of axon growth and bundling, with high RNA expression in brain tissue, and its inactivation promotes motor axon fasciculation and renewal in humans and mice [75]. Moreover, FGG, which had the second highest PC1 loading, was also high in serum from PD patients in a previous study [76], and in the rat model, its protein expression in the hippocampus and striatum was higher in PD rats than in healthy Sprague–Dawley rats [77]. FGG, FGA, and FGB physically interact with each other and are involved in stabilizing blood clots. Plasma fibrinogen was also elevated in PD patients in elderly Japanese-American men [78]. By single-cell RT-PCR analysis, amyloid-β + /α-synuclein + B cells favored the use of IGKV1 [79]. CA1 dysfunction impairs cognition function such as long-term synaptic transformation, memory storage, mental retardation and is associated with Alzheimer’s disease [80–82]. A PRSS3 gene was related to some non-synonymous variants in PD loci for axial impairment [83]. In PD, α-synuclein gene (SNCA) and heme metabolism genes BLVRB [84], HBD [85] and MPO [86–88] form a block of tightly correlated gene expression in human blood [84]. Morphologically, platelet abnormality in neurodegenerative conditions [89] might be linked with the downregulated platelet-associated proteins, PPDP [90, 91], GP5 [92], PF4 [93] and PF4V1 in PD. ER stress and neurological inflammation were related to THBS1/TGF-β signaling in PD [94], and known neuroinflammatory markers, AZU1 [95] and TREML1 [96, 97], were also lowered in PD.

### Brain-derived blood exosome biomarkers in the disease groups

**Fig. 5:**
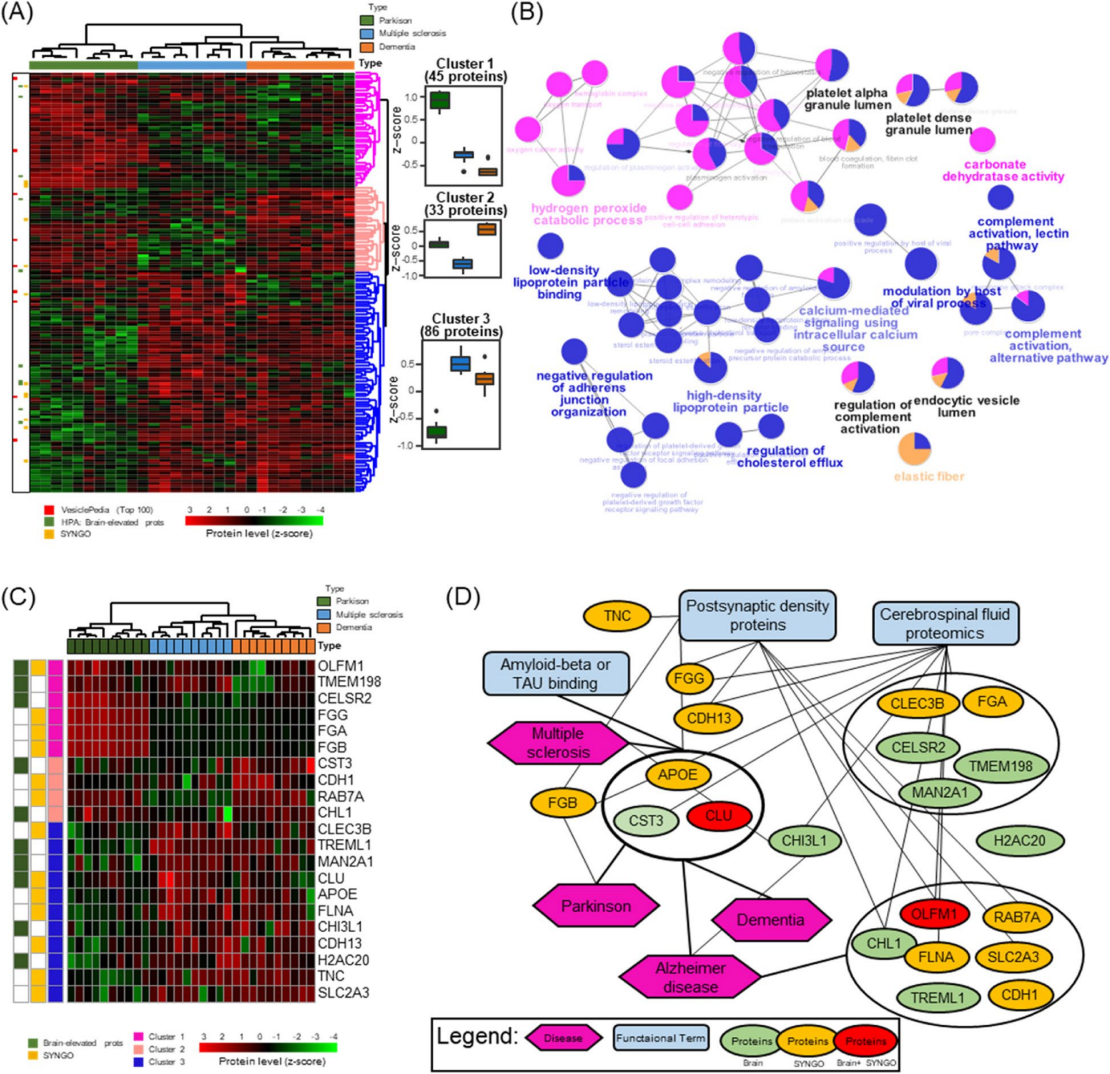
Hieratical clustering functional annotation of proteins with *p*-values less than 0.05 in one-way ANOVA tests. **A** A heatmap of 164 proteins by Euclidean distances with complete linkage has three protein clusters. **B** Functional GO network displaying groupings of biological process terms enriched in cluster 1 (magenta), cluster 2 (apricot), and cluster 3 (blue). Row labels consist of the top 100 proteins in Vesiclepedia (red bar), brain-elevated proteins in the Human Protein Atlas (green bar), and proteins annotated in Synaptic Gene Ontologies (SYNGO; yellow bar). **C** A truncated heatmap of 21 proteins related to neuronal mechanisms. **D** Association between the proteins (green circle: brain-elevated proteins, yellow: SYNGO, red: both) and disease (magenta hexagon) or functional annotation terms (blue square).

The 164 DAPs among the three groups were analyzed by one-way ANOVA with Benjamini–Hochberg correction (adjusted *P* < 0.05), annotated in tissue specificity and subcellular location in UniProt, and manually searched in literature for brain disease by PubTator Central [98] (Supplementary Table S6). Among them, the 52 DAPs (~ 31.71%) were secreted into blood and the 20 DAPs (~ 12.20%) were specifically expressed in brain. Interestingly, the 101 DAPs (~ 61.59%) were confirmed in the literature with at least the three neurodegenerative disease groups, PD with 53 proteins, MS with 62 proteins and dementia with 81 proteins, indicating that the exosomal proteins identified here deeply explained neurodegenerative conditions. By the hierarchical cluster analysis based on the protein abundance, we identified three protein clusters for finding out the disparate function between the disease groups (Fig. 5a). The cluster #1 proteins were most abundant in PD compared to the other two groups, and some were highly involved in the "hydrogen peroxide catabolic process," "positive regulation of heterotypic cell − cell adhesion," and "carbonate dehydratase activity." The proteins in cluster #2 were most abundant in dementia, followed by PD and MS, and were closely associated with "elastic fiber." Finally, protein cluster #3 was less abundant in PD compared to the other two groups. Proteins in cluster #3 were closely associated with "low- or high-density lipoprotein particle," "regulation of cholesterol efflux," "complement activation," and "modulation by host of viral process." The proteins in all three clusters were commonly associated with "platelet alpha or dense granule lumen," "endocytic vesicle lumen," and "regulation of complement activation" (Fig. 5b). The connection with proteins and functional terms was described in Supplementary Table S7. Furthermore, we found 21 proteins highly expressed in the brain in the HPA [71] or involved in neuron synapses by SYNGO [70] that could be used to identify exosome proteins derived from brain tissue (Table 1, Fig. 5c, and Supplementary Fig. S7). APOE, CST3, and CLU are known to be mainly involved in all three neurodegenerative diseases and, in particular, have a function of binding to amyloid-β or tau protein (Fig. 5d), indicating an association with features of neurodegenerative diseases due to protein aggregation of misfolded proteins [99]. More than half of the 21 proteins overlapped with previous reported CSF proteomic results, seven of which were in the postsynaptic density from EnrichR [67]. It means that some proteins pass through the BBB or blood–CSF barrier in the form of exosomes and are measured in the blood, which contributes to the classification of the three neurodegenerative diseases. Moreover, the average silhouette width of sample groups was 0.53, indicating that is more cohesively clustered than those of sex (0.21) or age (0.15) (Supplementary Fig. S8 and Supplementary Table S1).

**Table 1:** The differential abundant brain-associated proteins among three samples groups

**Uniprot Accession**	**Species**	**Gene**	**Protein name**	**SYNGO **[70]	**Brain-elevated proteins**	**Cluster number**
P02649	HUMAN	APOE	Apolipoprotein E	Y	N	Cluster 3
P12830	HUMAN	CDH1	Cadherin-1	Y	N	Cluster 2
P55290	HUMAN	CDH13	Cadherin-13	Y	N	Cluster 3
Q9HCU4	HUMAN	CELSR2	Cadherin EGF LAG seven-pass G-type receptor 2	N	Y	Cluster 1
P36222	HUMAN	CHI3L1	Chitinase-3-like protein 1	N	Y	Cluster 3
O00533	HUMAN	CHL1	Neural cell adhesion molecule L1-like protein	N	Y	Cluster 2
P05452	HUMAN	CLEC3B	Tetranectin	Y	N	Cluster 3
P10909	HUMAN	CLU	Clusterin	Y	Y	Cluster 3
P01034	HUMAN	CST3	Cystatin-C	N	Y	Cluster 2
P02671	HUMAN	FGA	Fibrinogen alpha chain	Y	N	Cluster 1
P02675	HUMAN	FGB	Fibrinogen beta chain	Y	N	Cluster 1
P02679-2	HUMAN	FGG	Isoform Gamma-A of Fibrinogen gamma chain	Y	N	Cluster 1
P21333	HUMAN	FLNA	Filamin-A	Y	N	Cluster 3
Q16777	HUMAN	H2AC20	Histone H2A type 2-C	N	Y	Cluster 3
Q16706	HUMAN	MAN2A1	Alpha-mannosidase 2	N	Y	Cluster 3
Q99784	HUMAN	OLFM1	Noelin	Y	Y	Cluster 1
P51149	HUMAN	RAB7A	Ras-related protein Rab-7a	Y	N	Cluster 2
P11169	HUMAN	SLC2A3	Solute carrier family 2, facilitated glucose transporter member 3	Y	N	Cluster 3
Q66K66	HUMAN	TMEM198	Transmembrane protein 198	N	Y	Cluster 1
P24821	HUMAN	TNC	Tenascin	Y	N	Cluster 3
Q86YW5	HUMAN	TREML1	Trem-like transcript 1 protein	N	Y	Cluster 3

## Discussion

Exosomes are submicron bioparticles enclosed by a lipid bilayer membrane and released from various cells. The molecular contents (lipids, RNAs, and proteins) of these bioparticles reflect the origin of the cell. Exosomes have been isolated from most biological fluids, including saliva, urine, and blood [17–19]. However, despite important technical developments, the isolation and analysis of exosomes from clinical specimens have been hampered by several limitations, including the requirement of expensive and scale-up equipment as well as laborious and time-consuming procedures. Hence, accurate and convenient techniques are urgently needed to isolate exosomes with increased purity and yield. Exosomes have the ability to cross the BBB, and it is now known that particles produced by central nervous system cells can circulate in the blood, and exosomes from various origins can enter the central nervous system [100]

Transferrin receptor participates in the mechanism of exosome transport through BBB. It is worth mentioning that blood plasma contains a high amount of transferrin, which mainly binds to transferrin receptor on the surface of cerebral vascular endothelial cells as well as blood-derived exosomes [101].

Moreover, the transferrin receptor is a widely validated and utilized biomarker receptor for the receptor-mediated delivery of therapeutics across the BBB [102]. Furthermore, transferrin receptor-mediated brain delivery has been applied to various therapeutics, including chitosan nanospheres [103] and liposomes [104, 105]. Hence, in this study, to specifically isolate brain-derived exosomes from blood, we report an MTNs assay based on the conjugation of transferrin as a ligand to MNPs to isolate exosomes through the ligand − receptor interaction of transferrin and transferrin receptor for retrieving a high abundance of blood-derived exosomes with high biological safety.

Several challenges for the isolation of exosomes have been addressed in this study. First, the MTNs were synthesized via a one-pot approach (Fig. 1) to overcome the time-consuming and laborious issues. The MTNs assay is simple and rapid (< 35 min) and does not require any antibody or centrifugation. The synthesized MTNs could efficiently capture exosomes while maintaining their biological functions. Second, in contrast to UC and TEI assays, ARF6 (a microvesicle related protein) was not detected in the exosomes isolated by the MTNs assay (Fig. 3d). These data suggest the possibility that the MTNs bind to the small EVs (exosomes) rather than large EVs (microvesicles). The MTNs assay might selectively capture exosomes. Despite this finding, further research is required to assess whether EV subpopulations can be isolated using MTNs alongside these other assays. Third, we validated that MTNs can be used to isolate exosomes from serum samples of patients with PD, MS, and dementia. The isolated exosome proteins were identified via LC–MS/MS to show distinct patterns for each disease. According to the proteomic results, we found 21 proteins highly expressed in neurons and implicated in neurodegenerative diseases (Fig. 5c). Literature searches related to brain disease were performed for the 21 proteins. Among the proteins, APOE, CLU, CHL1, SLC1A3, RAB7A, and CST3 were related to the protein aggregation of misfolded proteins—causative agents and symptomatic of neurodegenerative diseases—such as α-synuclein in PD, and amyloid-β and tau in dementia [99]. Recently, neurodegeneration in MS has also been related to the aggregation of the presynaptic scaffolding protein, Bassoon [106]. Apolipoproteins, such as APOE and APOJ, also known as CLU, can form complexes with amyloid-β, regulating its clearance across the BBB [107]. The type of APOE allele is a well-known genetic risk factor for dementia [108], PD [109] and MS [110], and people with the APOE ε4 allele are at increased risk for all three diseases. Quantitative changes in CLU in the brain, CSF, or blood associated with PD [111, 112], dementia [113–115], and MS [116]. Moreover, CHL1 is a neural cell adhesion molecule and a close homolog of BACE1, the rate-limiting enzyme for the production of β-amyloid peptide linked to Alzheimer's disease (AD) [117]. SLC2A3 (GLUT3) also affects β-amyloid peptide production [118], and SLC2A3 rs12842 polymorphism has a strong inverse association with the risk of AD [119]. RAB7A, which regulates tau secretion, was identified through deletion in vivo in mouse experiments [120, 121]. CST3 is abundant in the CSF and implicated in cell signaling, inflammation, and neuronal cell death [122]. It is highly present in the serum of patients with PD [123] and has the potential for use as a therapeutic agent [124]. Furthermore, some researchers are developing a peptide therapy for biopanning on the TNC protein [125, 126] that is highly expressed in chronic MS lesions or numerous neurodegenerative diseases [127]. CHI3L1 has been studied as a biomarker candidate for the early screening of neuritis and examination of AD [128] and is high in the serum of patients with AD [129] and the CFS of patients with MS [130]. Fourth, this study merits attention in that the results suggest novel blood-based disease biomarkers in neurodegenerative diseases. Due to the progressive and irreversible nature of neurodegenerative diseases, biomarkers that predict and monitor the disease course are necessary. Currently, imaging biomarkers, such as magnetic resonance imaging (MRI) and positron emission tomography (PET) biomarkers, are used to diagnose and monitor neurodegenerative diseases. However, these tests are prone to be inconvenient and too expensive to perform regularly (e.g., once every several months). In addition, their changes may not be sensitive enough to detect subclinical changes in the brain at the molecular level. Biomarkers in CSF may be more sensitive to reflect subclinical brain changes than imaging biomarkers, but the invasiveness of the test prevents CSF biomarkers from being widely used. Blood biomarkers of brain-derived exosomes can avoid these shortcomings and thus will be clinically useful. It should also be emphasized that this study suggests brain-derived blood proteins as specific biomarkers for stratifying neurodegenerative diseases. This is remarkable in that current promising blood protein biomarkers are mainly biomarkers that reflect neurologic damage and/or degeneration sensitively, not specific biomarkers that discriminate a particular neurologic disease from the others. For instance, the increase in blood levels of NfL, which reflects the disease course of MS sensitively [131], does not necessarily indicate the relapse or aggravation of MS because an NfL increase can be induced by various neurologic conditions [6, 132]. This non-specificity is also shown similarly in the case of amyloid-β or tau proteins because these proteins could increase in all the conditions of neurodegeneration, not only in dementia but in PD and even amyotrophic lateral sclerosis [133–135]. Therefore, the specific characteristic of brain-derived EV protein biomarkers with distinct patterns for each neurological disease may be useful in clinical practice, complementing currently available sensitive but non-specific blood biomarkers.

Despite the advantages of the MTNs assay for exosome isolation from clinical specimens, there are several issues to be addressed in future studies. First, detailed information, such as disease severity and medication information, was not evaluated. The possibility that patterns of blood exosome proteins have been grouped by factors other than intrinsic disease characteristics cannot be excluded. Age and the female proportion were particularly different across the analyzed disease groups. Thus, our findings should be interpreted cautiously, and future confirmational studies with detailed information on the patients are warranted, given the pilot nature of this study. Despite the above concerns, the sample groups (PD, MS, dementia) are more cohesively clustered than sex or age in the PC1 and PC2 of exosome proteins (Supplementary Fig. 8). Second, the diagnostic value of exosome proteins was not verified in the independent disease cohort. Third, the origin of exosomes in the blood is uncertain, and the purity of neural cell-derived exosomes is not identified. If some membrane proteins highly contained in neural-cell-derived exosomes, such as exosome membrane proteins, for example, transferrin, are covalently linked to MTNs, then the purity of neural-cell-derived exosomes in the blood is expected to be improved. Despite that some serum proteins, such as albumin and immunoglobulins, contaminate the exosome proteome data from LC–MS/MS in neurodegenerative diseases, we found three cytokines, namely CCL14, CC18, and TNFRSF1A, which were difficult to quantify due to low concentrations in the previous serum proteome studies [136], but were quantified using the MTNs assay. Thus, this assay is useful for the discovery of low-abundant protein biomarkers in blood specimens without the abundance-bias issue. Fourth, the brain-derived blood exosomes identified in this study must be validated as biomarkers in a large cohort clinical study. Fifth, the population of exosomes present in the blood is very heterogenous because circulating vesicles are released by different types of cells, either circulating blood cells or cells having contact with circulation (e.g., endothelial cells). Contamination of isolated exosomes with non-exosomal particles, such as apoptotic bodies, small apoptotic vesicles, exomeres, and lipoproteins, can cause wrong conclusions about biological activities of the obtained exosomes and therefore should be avoided [137]. The main limitation in EV (exosomes) proteomics and lipidomics is contamination with other types of EVs (exosomes), thus the purity largely depends on the isolation techniques used. For this reason, it is currently impossible to separate EVs (exosomes) based on their biogenic origins, and thus the analysis of bona fide EVs (exosomes) is challenging. It is necessary for the next step to isolate and analyze CNS cell-specific exosomes directly derived from neurons and glial cells. Attempts to isolate CNS cell-specific exosomes are underway [138], and these techniques would be able to bind successfully to our proposed MTN analysis. Meanwhile, biomarkers do not necessarily have to be involved in the main pathogenesis to reflect disease processes. Thus, exosomes derived from non-brain cells may also have a role as a specific disease biomarker for neurological disorders. However, this potential should be investigated and confirmed with a larger number of blood samples with a context of validating biomarkers positively correlated with disease activity and severity within a specific disease category. Nevertheless, the MTNs assay offers a promising avenue for exosome isolation from biofluid specimens. Implementation of a high-throughput proteomics platform with MTNs for exosome protein detection may clarify the biomolecular sign associated with the disease.

## Conclusion

We demonstrated the applicability of the MTNs assay to the isolation of blood-derived exosomes, thereby providing a convenient approach for the rapid and affordable isolation of clinically applicable exosomes from body fluids for noninvasive diagnosis. We envision a great potential for MTNs in EV-related applications, such as stem cell treatments, transplantations, immune-based treatments, and theranostics in neurological diseases.

## Supplementary Information

**Additional file 1**: **Supplementary Fig. S1.** Characterization of MTNs assay. (a) Washing buffer testing either with ethanol or distilled water for effectiveness on the Fe3O4@SiO2-NH2 MNPs. (b) Testing of various transferrin concentrations and determined the optimal concentration via the zeta potential analysis. (c) Performance of MTNs depends on incubation time (24 h or 3 h) with either 10 mL HCT-116 cell culture medium (1-2 & 5-6) or 500 µL human normal serum sample (3-4 & 7-8) using Coomassie blue staining. (d) Western blot result from 24 h incubation with either 10 mL HCT-116 cell culture medium (1-2) or 500 µL human normal serum sample (3-4). **Supplementary Fig. S2.** Characterization of the isolated exosomes. (a−c) Representative SEM images and NTA of the exosomes isolated from colon cancer CCM using (a and b) MTNs, (c and d) UC, and (e and f) TEI. **Supplementary Fig. S3.** Validation of exosome isolation. (a and b) Representative (a) SEM image and (b) zeta-potential of the exosomes isolated from colon cancer CCM using MTNs. Supplementary Fig. S4. Flowchart of statistical analysis and batch effect correction (a) From the first 746 quantified proteins, 550 proteins that were quantified at least 70% in at least one of the three groups were selected, log_2_-transformed, and normalized by the width adjustment method. Then, missing values were estimated from a normal distribution with an area of 0.3 minus 1.8 from the protein distribution for each sample, and batch 1 and batch 2 were adjusted for the batch effect with the protein average. Principal component analysis plot (b) before batch correction and (c) after batch correction. Circle (batch 1 samples), filled rectangle (batch 2 samples), green (Parkinson’s disease), blue (multiple sclerosis), orange (dementia). **Supplementary Fig. S5.** Distribution of normalized protein abundances based on label-free quantification. (a) Mapping proteins to exosomes public database, Vesiclepedia. Exosome top 100 proteins are highlighted in red. Proteins belonging to Vesiclepedia are shown in blue. The remaining proteins are shown in gray. (b) Mapping proteins to brain-elevated protein in the Human Protein Atlas. Brain-elevated proteins are highlighted in red. The remaining proteins are shown in gray. **Supplementary Fig. S6.** Boxplots of 20 proteins with top 10 and bottom 10 proteins with loadings of principal component 1. (a) Top 10 proteins (b) Bottom 10 proteins. Green (Parkinson’s disease), blue (multiple sclerosis), orange (dementia); * *p* < 0.05, ** *p* < 0.01, *** *p* < 0.001, **** *p* < 0.0001, n.s., not significant. **Supplementary Fig. S7.** Boxplots of 21 proteins highly expressed in the brain in the HPA or annotated in SYNGO. Green (Parkinson’s disease), blue (multiple sclerosis), orange (dementia); * *p* < 0.05, ** *p* < 0.01, *** *p* < 0.001, **** *p* < 0.0001, n.s., not significant. **Supplementary Fig. S8.** Principal component analysis (PCA) of plasma exosome proteins and assessment of the relative quality of clustering by silhouette method. (a) PCA by the three sample groups (Parkinson’s disease (PD; *N* = 10), multiple sclerosis (MS; *N* = 10), dementia (Dem; *N* = 10)) and silhouette plot of the groups. (b) PCA by sex (female (*N* = 16) and male (*N* = 14)) and silhouette plot of sex. (c) PCA by age groups based on age 65 (age>65 (*N* = 15) and age≤65 (*N* = 15)) and silhouette plot of the age groups. **Supplementary Table S1.** Baseline characteristics of the patients.

**Additional file 2:** **Supplementary Table S2**. Quantified 746 raw protein abundance in 30 samples in three groups, dementia, Parkinson, and multiple scleroisis.

**Additional file 3:** **Supplementary Table S3.** Normalized 550 protein abundances in 30 samples in three groups, dementia, Parkinson, and multiple scleroisis.

**Additional file 4:** **Supplementary Table S4.** Functional annotation of normalized 550 proteins.

**Additional file 5:** **Supplementary Table S5**. The loading and reference of PC1-highly contributed 20 proteins.

**Additional file 6:** **Supplementary Table S6.** Differential abundant 164 serum exosome proteins among three groups, dementia, Parkinson, and multiple scleroisis (FDR q < 0.05). The table included the information about cluster number, ANOVA p-values, ANOVA q-values, SYNGO, brain-elevated genes in Human Protein Atlas (HPA) and top 100 exosome proteins in Vesiclepedia, subcelluarl location in UniProt, tissue specificity in UniProt, and pubmed ids.

**Additional file 7:** **Supplementary Table S7.** Functional GO terms and exosomal protein network list in the cluster #1, #2 and #3.

## Data Availability

Data sharing is not applicable to this article.

## Abbreviations

AD: Alzheimer’s disease
APTES: 3-Aminopropyl triethoxysilane
BBB: Blood − brain barrier
CCM: Cell culture medium
CSF: Cerebrospinal fluid
DAP: Differentially abundant protein
DMEM: Dulbecco’s modified Eagle medium
DW: Distilled water
EV: Extracellular vesicle
FA: Formic acid
FBS: Fetal bovine serum
FDR: False discovery rate
FTIR: Fourier-transform infrared
GA: Glutaraldehyde
GO: Gene ontology
LC–MS: Liquid chromatography − mass spectrometry
MNP: Magnetic nanoparticle
MS: Multiple sclerosis
MS/MS: Tandem mass spectrometry
MTN: Magnetic transferrin nanoparticle
NTA: Nanoparticle tracking analysis
PBS: Phosphate-buffered saline
PCA: Principal component analysis
PD: Parkinson’s disease
RT: Room temperature
RT-Qpcr: Quantitative reverse transcription PCR
SEM: Scanning electron microscopy
TEI: Total exosome isolation
TEM: Transmission electron microscopy
UC: Ultracentrifugation
UV–Vis: Ultraviolet–visible
WB: Western blotting
