# Supplementary material for: Magnetic transferrin nanoparticles (MTNs) assay as a novel isolation approach for exosomal biomarkers in neurological diseases
Source: Biomater Res. 2023 Feb 16;27:12. doi: 10.1186/s40824-023-00353-2 (PMC9936675; doi:10.1186/s40824-023-00353-2)
Supplement: Supplementary file 1 — Additional file 1: Supplementary Fig. S1. Characterization of MTNs assay. (a) Washing buffer testing either with ethanol or distilled water for effectiveness on the Fe3O4@SiO2-NH2 MNPs. (b) Testing of various transferrin concentrations and determined the optimal concentration via the zeta potential analysis. (c) Performance of MTNs depends on incubation time (24 h or 3 h) with either 10 mL HCT-116 cell culture medium (1-2 & 5-6) or 500 µL human normal serum sample (3-4 & 7-8) using Coomassie blue staining. (d) Western blot result from 24 h incubation with either 10 mL HCT-116 cell culture medium (1-2) or 500 µL human normal serum sample (3-4). Supplementary Fig. S2. Characterization of the isolated exosomes. (a−c) Representative SEM images and NTA of the exosomes isolated from colon cancer CCM using (a and b) MTNs, (c and d) UC, and (e and f) TEI. Supplementary Fig. S3. Validation of exosome isolation. (a and b) Representative (a) SEM image and (b) zeta-potential of the exosomes isolated from colon cancer CCM using MTNs. Supplementary Fig. S4. Flowchart of statistical analysis and batch effect correction (a) From the first 746 quantified proteins, 550 proteins that were quantified at least 70% in at least one of the three groups were selected, log2-transformed, and normalized by the width adjustment method. Then, missing values were estimated from a normal distribution with an area of 0.3 minus 1.8 from the protein distribution for each sample, and batch 1 and batch 2 were adjusted for the batch effect with the protein average. Principal component analysis plot (b) before batch correction and (c) after batch correction. Circle (batch 1 samples), filled rectangle (batch 2 samples), green (Parkinson’s disease), blue (multiple sclerosis), orange (dementia). Supplementary Fig. S5. Distribution of normalized protein abundances based on label-free quantification. (a) Mapping proteins to exosomes public database, Vesiclepedia. Exosome top 100 proteins are highlighted in re [file 40824_2023_353_MOESM1_ESM.docx]

**Supplementary**

**Magnetic transferrin nanoparticles (MTNs)** **assay as a novel isolation approach for exosomal biomarkers in neurological diseases**

Yoon Ok Jang^1,#^, Hee-Sung Ahn^2,#^, Thuy Nguyen Thi Dao^1^, JeongYeon Hong^3,4^, Wangyong Shin^5^, Young-Min Lim^5^, Sun Ju Chung^5^, Jae-Hong Lee^5^, Huifang Liu^1^, Bonhan Koo^1^, Myoung Gyu Kim^1^, Kyunggon Kim^3,4,^*, Eun-Jae Lee^5,^*, Yong Shin^1,^*

^1^Department of Biotechnology, College of Life Science and Biotechnology, Yonsei University, Seoul 03722, Republic of Korea

^2^Department of Convergence Medicine, Asan Medical Center, Seoul 05505, Republic of Korea

^3^Asan Institute for Life Sciences, Asan Medical Center, Seoul 05505, Republic of Korea

^4^Department of Biomedical Sciences, University of Ulsan College of Medicine, Seoul 05505, Republic of Korea

^5^Department of Neurology, Asan Medical Center, University of Ulsan College of Medicine, Seoul 05505, Republic of Korea

^#^These authors (Y.O.J. and H.-S.A.) equally contributed to this study.

*Corresponding authors: Email: [shinyongno1@yonsei.ac.kr](mailto:shinyongno1@yonsei.ac.kr) (Y. Shin), eunjae.lee@amc.seoul.kr (E-J. Lee), [kimkyunggon@gmail.com](mailto:kimkyunggon@gmail.com) (K. Kim)

Supplementary Fig. S1-8

Supplementary Table S1 and Table S2-S7 (as separate excel files)


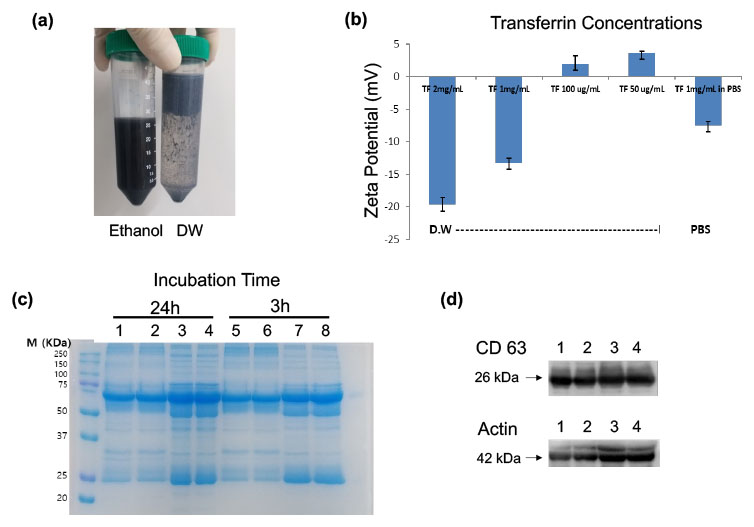


**Supplementary Fig. S1** Characterization of MTNs assay. (**a**) Washing buffer testing either with ethanol or distilled water for effectiveness on the Fe3O4@SiO2-NH2 MNPs. (**b)** Testing of various transferrin concentrations and determined the optimal concentration via the zeta potential analysis. (**c**) Performance of MTNs depends on incubation time (24 h or 3 h) with either 10 mL HCT-116 cell culture medium (1-2 & 5-6) or 500 µL human normal serum sample (3-4 & 7-8) using Coomassie blue staining. (**d)** Western blot result from 24 h incubation with either 10 mL HCT-116 cell culture medium (1-2) or 500 µL human normal serum sample (3-4).

**Supplementary Fig. S2** Characterization of the isolated exosomes. (**a**−**c**) Representative SEM images and NTA of the exosomes isolated from colon cancer CCM using (**a** and **b**) MTNs, (**c** and **d**) UC, and (**e** and **f**) TEI.


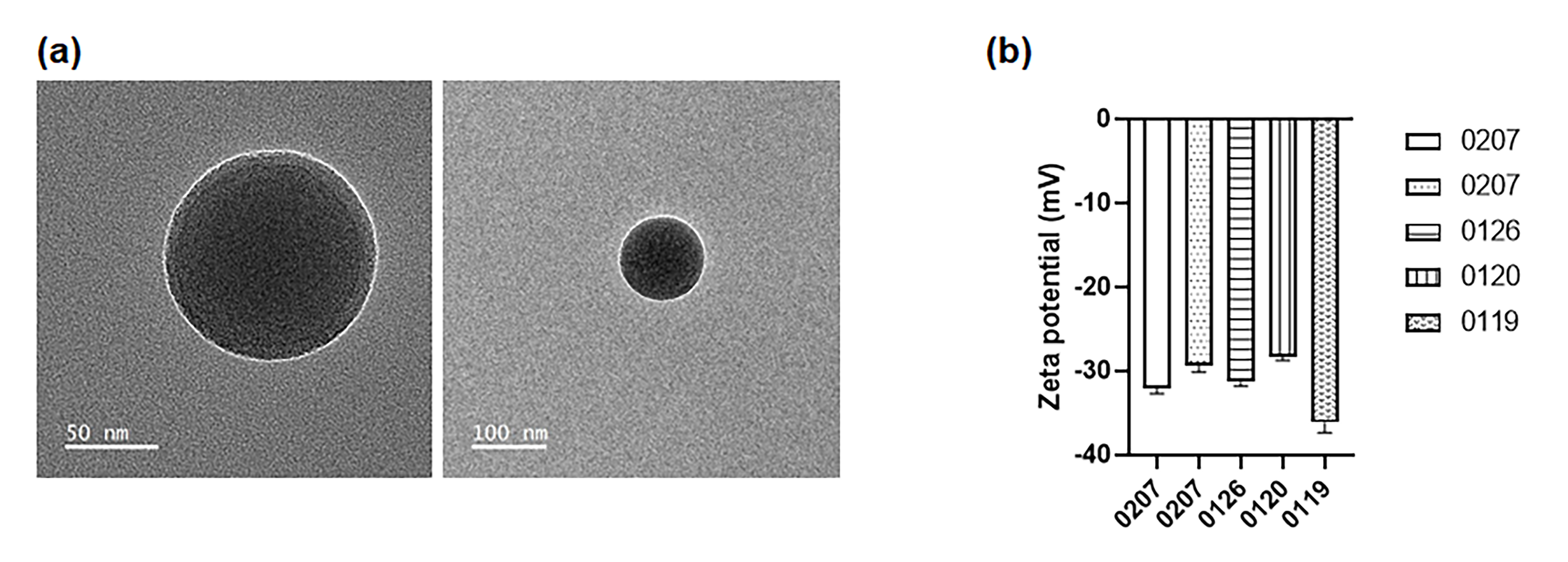


**Supplementary Fig. S3** Validation of exosome isolation. (**a** and **b**) Representative (**a**) SEM image and (**b**) zeta-potential of the exosomes isolated from colon cancer CCM using MTNs.

**
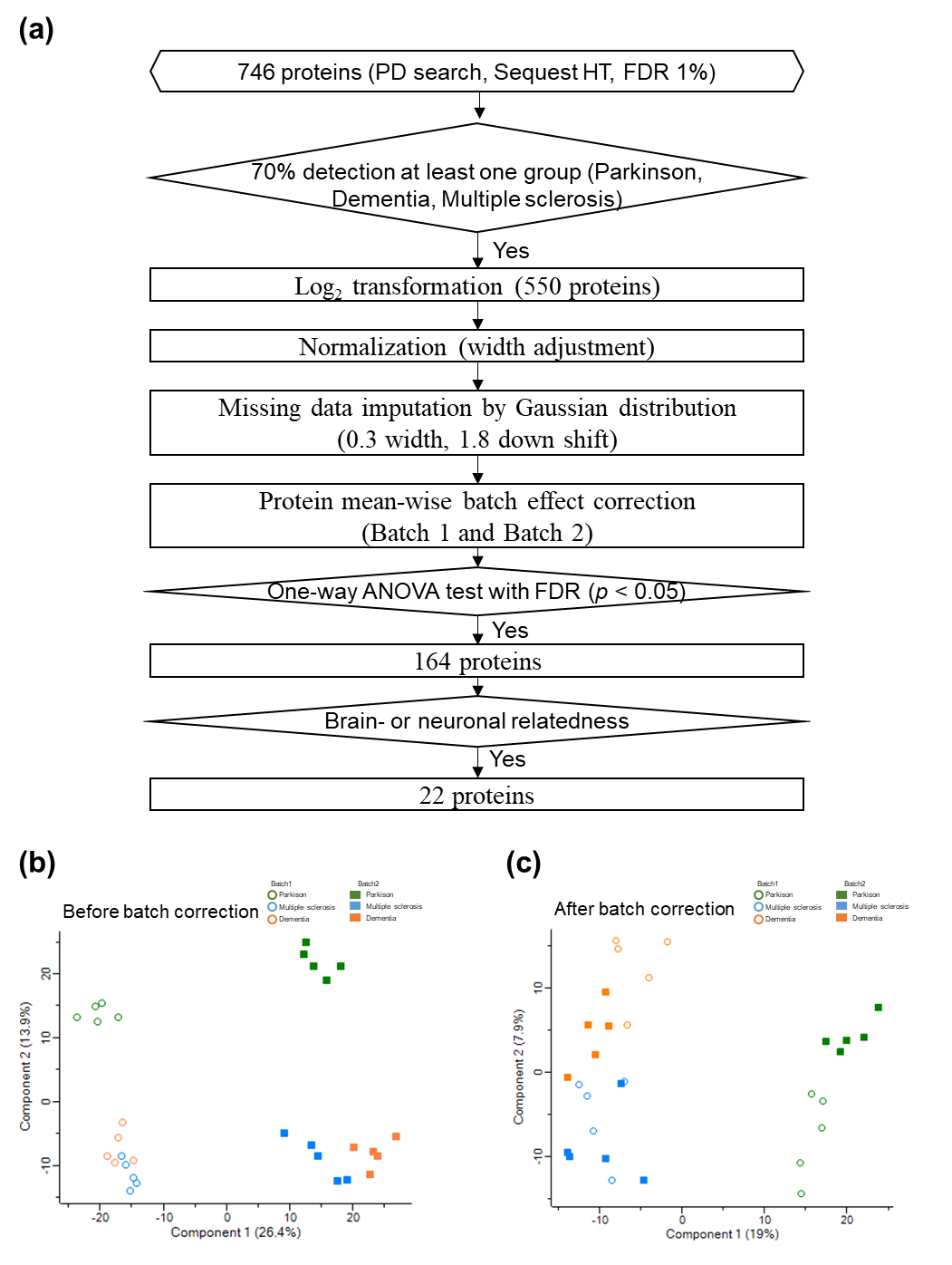
**

**Supplementary Fig. S4** Flowchart of statistical analysis and batch effect correction (**a**) From the first 746 quantified proteins, 550 proteins that were quantified at least 70% in at least one of the three groups were selected, log_2_-transformed, and normalized by the width adjustment method. Then, missing values were estimated from a normal distribution with an area of 0.3 minus 1.8 from the protein distribution for each sample, and batch 1 and batch 2 were adjusted for the batch effect with the protein average. Principal component analysis plot (**b**) before batch correction and (**c**) after batch correction. Circle (batch 1 samples), filled rectangle (batch 2 samples), green (Parkinson’s disease), blue (multiple sclerosis), orange (dementia).

**
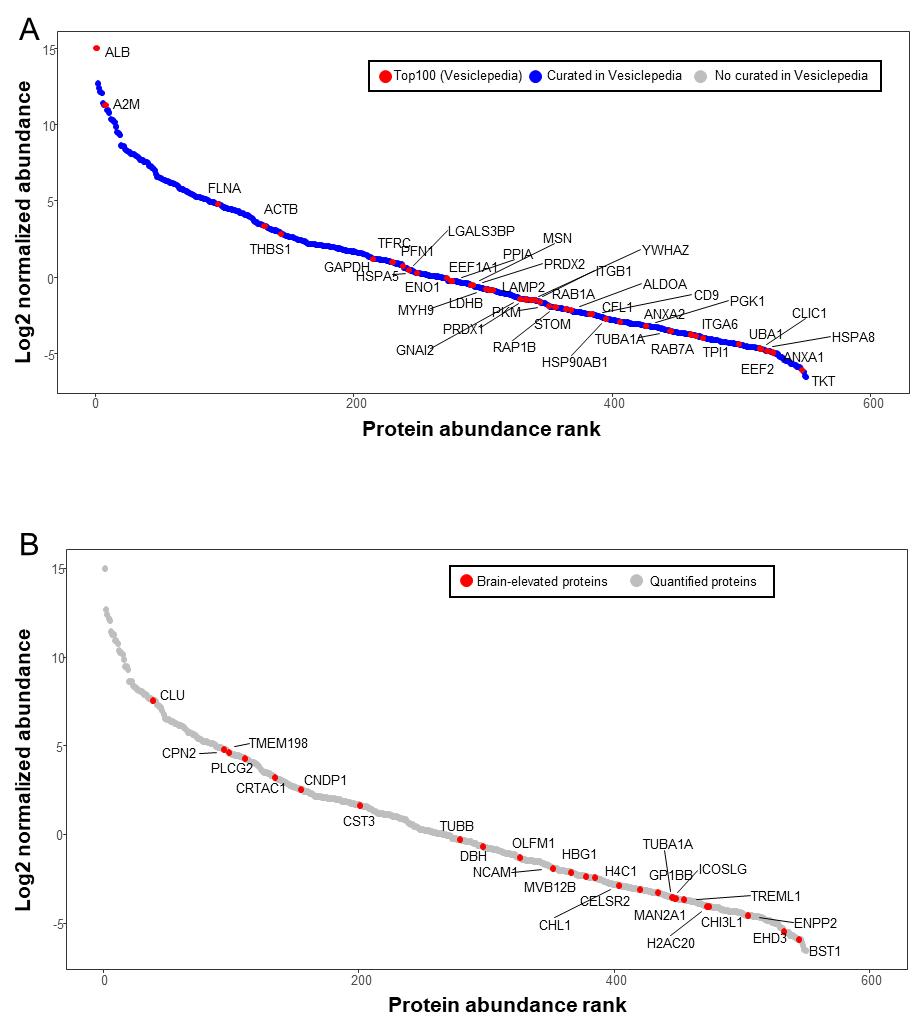
**

**Supplementary Fig. S5** Distribution of normalized protein abundances based on label-free quantification. (**a**) Mapping proteins to exosomes public database, Vesiclepedia. Exosome top 100 proteins are highlighted in red. Proteins belonging to Vesiclepedia are shown in blue. The remaining proteins are shown in gray. (**b**) Mapping proteins to brain-elevated protein in the Human Protein Atlas. Brain-elevated proteins are highlighted in red. The remaining proteins are shown in gray.


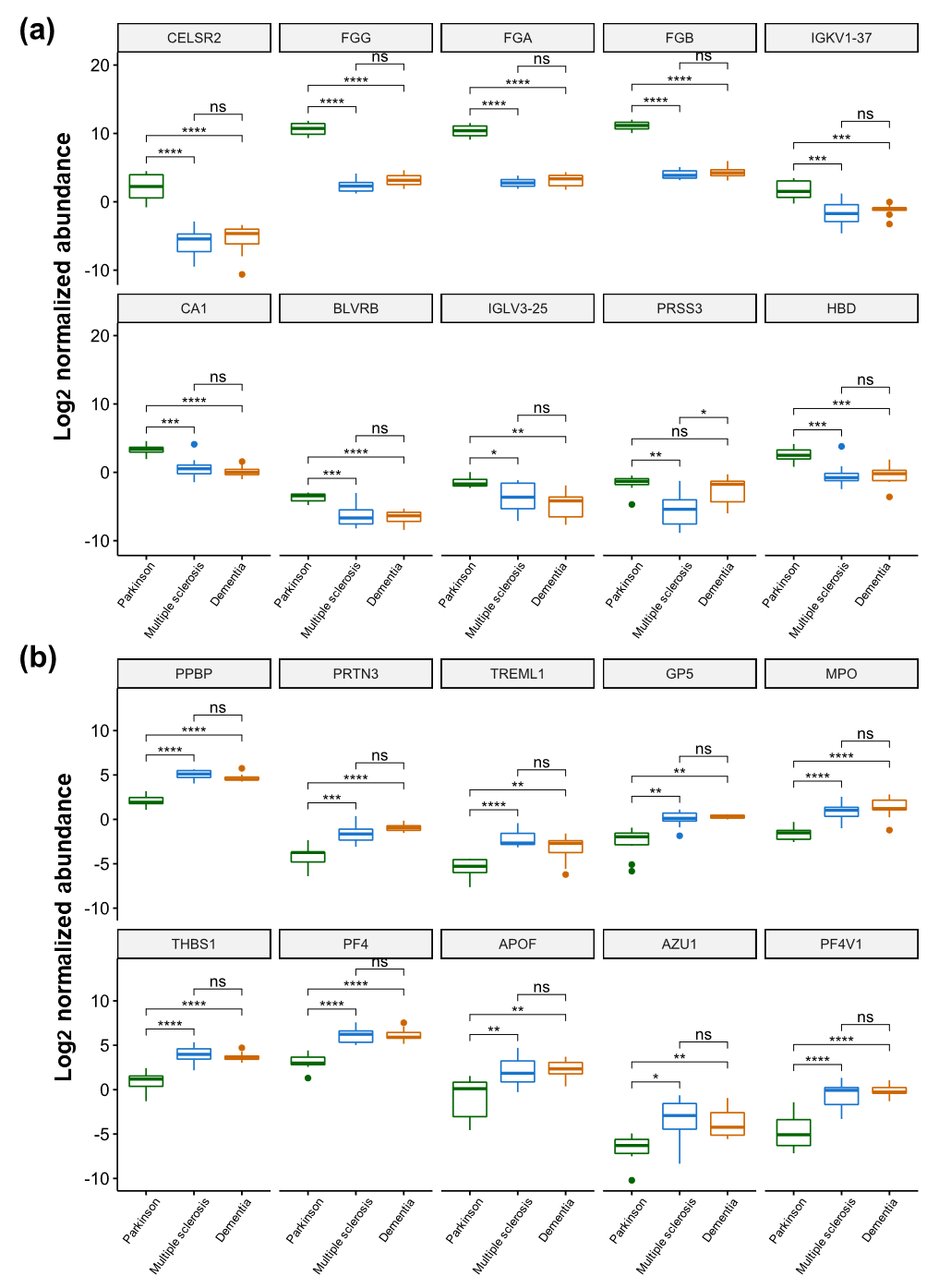


**Supplementary Fig. S6** Boxplots of 20 proteins with top 10 and bottom 10 proteins with loadings of principal component 1. (**a**) Top 10 proteins (**b**) Bottom 10 proteins. Green (Parkinson’s disease), blue (multiple sclerosis), orange (dementia); * *p* < 0.05, ** *p* < 0.01, *** *p* < 0.001, **** *p* < 0.0001, n.s., not significant.


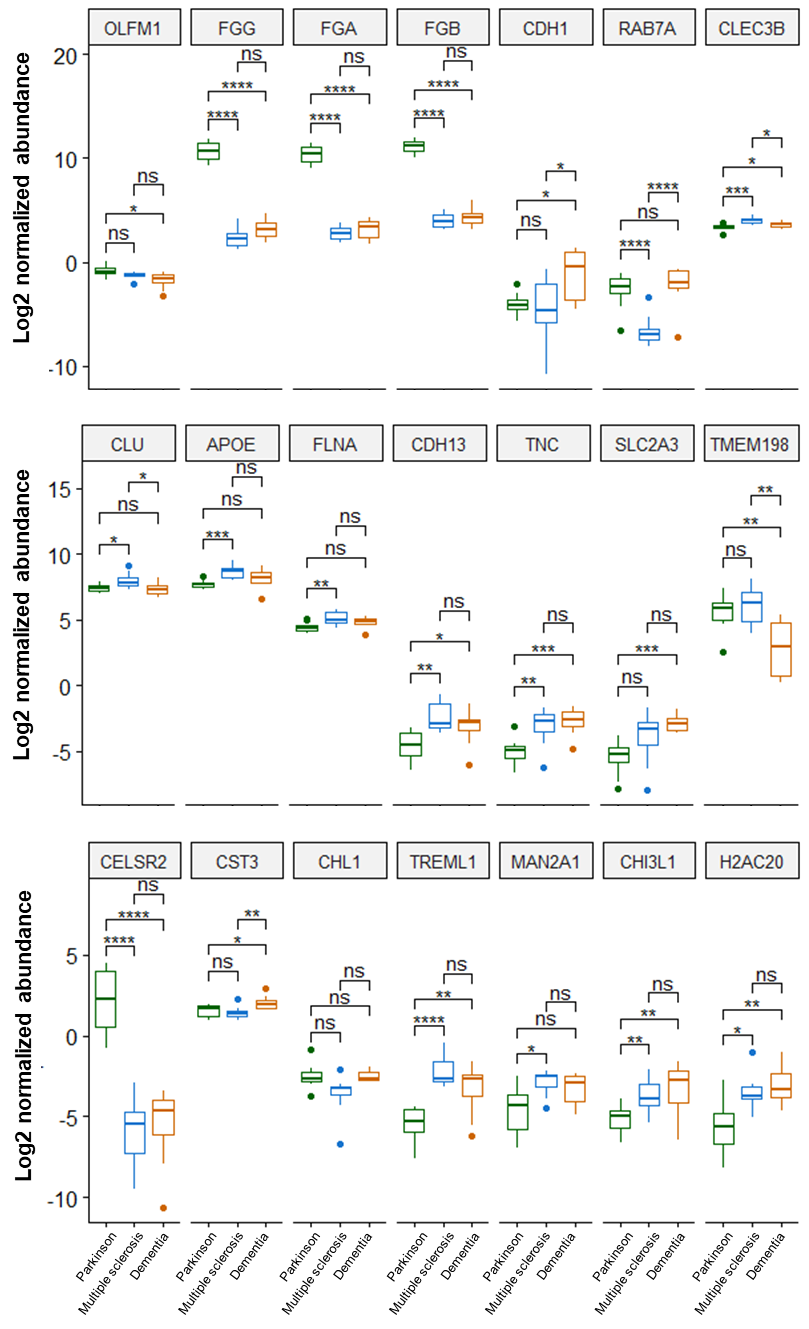


**Supplementary Fig. S7** Boxplots of 21 proteins highly expressed in the brain in the HPA or annotated in SYNGO. Green (Parkinson’s disease), blue (multiple sclerosis), orange (dementia); * *p* < 0.05, ** *p* < 0.01, *** *p* < 0.001, **** *p* < 0.0001, n.s., not significant.


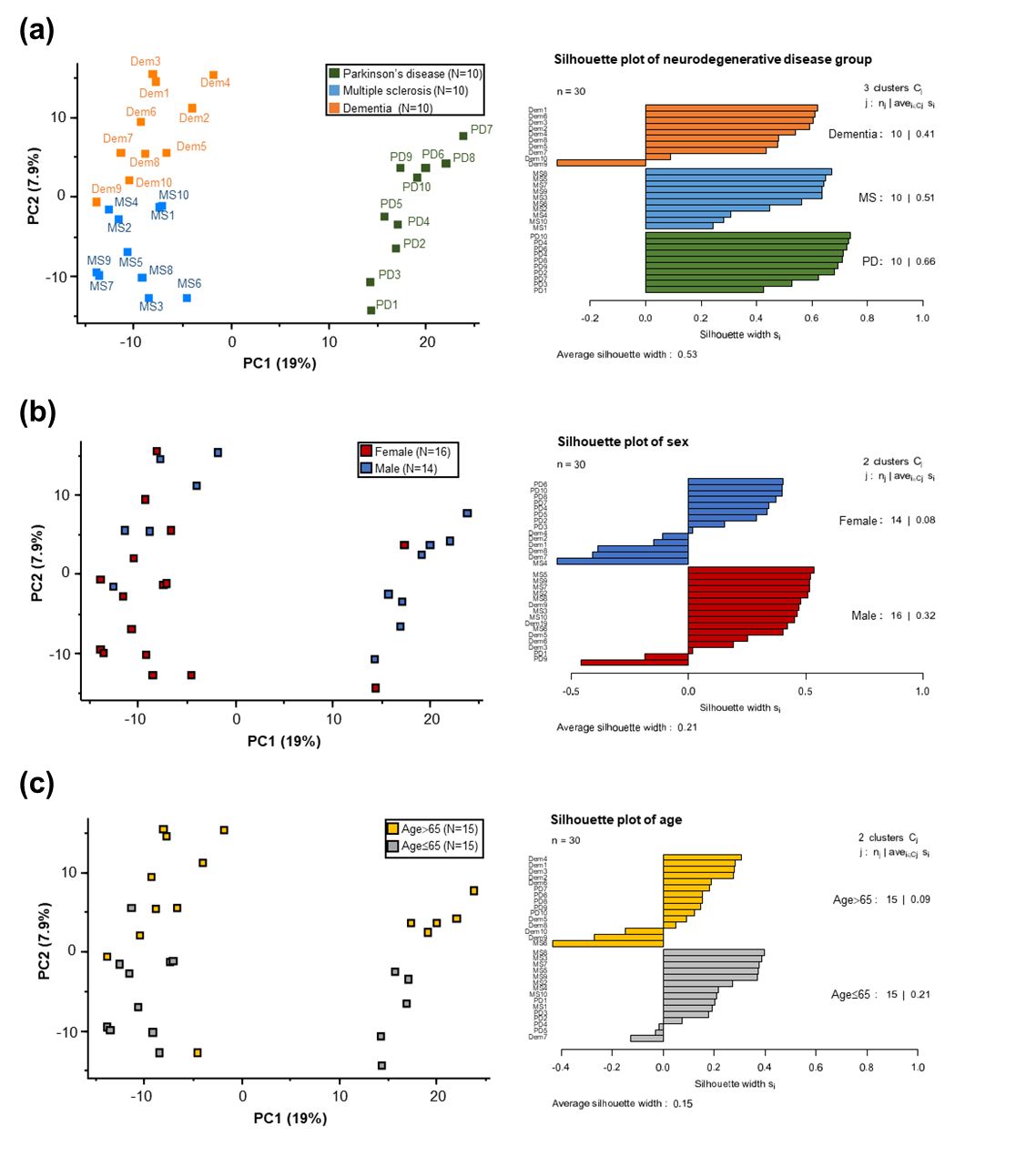


**Supplementary Fig. S8** Principal component analysis (PCA) of plasma exosome proteins and assessment of the relative quality of clustering by silhouette method. (**a**) PCA by the three sample groups (Parkinson’s disease (PD; N = 10), multiple sclerosis (MS; N = 10), dementia (Dem; N = 10)) and silhouette plot of the groups. (**b**) PCA by sex (female (N = 16) and male (N = 14)) and silhouette plot of sex. (**c**) PCA by age groups based on age 65 (age>65 (N = 15) and age≤65 (N = 15)) and silhouette plot of the age groups.

**Supplementary Table S1**. Baseline characteristics of the patients

|  | MS | Parkinson | Dementia |
| --- | --- | --- | --- |
|  | (n = 10) | (n = 10) | (n = 10) |
| Age – median (IQR) | 61 (25–75) | 65 (61–69) | 78 (71–86) |
| Female – n (%) | 9 (90%) | 2 (20%) | 5 (50%) |
| EDSS – median (IQR) | 4.0 (2.5–6.5) |  |  |
| HY stage – median (IQR) |  | 2.5 (2.4–2.6) |  |
| GDS – median (IQR) |  |  | 4 (3.0–4.0) |

EDSS, Expanded Disability Status Scale; GDS, Global Deterioration Scale; HY stage, Hoehn-Yahr stage; IQR, interquartile range; MS, multiple sclerosis.
